# Combining molecular dynamics simulations and scoring method to computationally model ubiquitylated linker histones in chromatosomes

**DOI:** 10.1371/journal.pcbi.1010531

**Published:** 2023-08-01

**Authors:** Kevin Sawade, Andreas Marx, Christine Peter, Oleksandra Kukharenko

**Affiliations:** 1 Department of Chemistry, University of Konstanz, Konstanz, Germany; 2 Theory Department, Max-Planck Institute for Polymer Research, Mainz, Germany; Stockholm University: Stockholms Universitet, SWEDEN

## Abstract

The chromatin in eukaryotic cells plays a fundamental role in all processes during a cell’s life cycle. This nucleoprotein is normally tightly packed but needs to be unpacked for expression and division. The linker histones are critical for such packaging processes and while most experimental and simulation works recognize their crucial importance, the focus is nearly always set on the nucleosome as the basic chromatin building block. Linker histones can undergo several modifications, but only few studies on their ubiquitylation have been conducted. Mono-ubiquitylated linker histones (HUb), while poorly understood, are expected to influence DNA compaction. The size of ubiquitin and the globular domain of the linker histone are comparable and one would expect an increased disorder upon ubiquitylation of the linker histone. However, the formation of higher order chromatin is not hindered and ubiquitylation of the linker histone may even promote gene expression. Structural data on chromatosomes is rare and HUb has never been modeled in a chromatosome so far. Descriptions of the chromatin complex with HUb would greatly benefit from computational structural data. In this study we generate molecular dynamics simulation data for six differently linked HUb variants with the help of a sampling scheme tailored to drive the exploration of phase space. We identify conformational sub-states of the six HUb variants using the sketch-map algorithm for dimensionality reduction and iterative HDBSCAN for clustering on the excessively sampled, shallow free energy landscapes. We present a highly efficient geometric scoring method to identify sub-states of HUb that fit into the nucleosome. We predict HUb conformations inside a nucleosome using on-dyad and off-dyad chromatosome structures as reference and show that unbiased simulations of HUb produce significantly more fitting than non-fitting HUb conformations. A tetranucleosome array is used to show that ubiquitylation can even occur in chromatin without too much steric clashes.

## Author summary

In eukaryotic cells the linker histones play a crucial role in the formation of higher order nucleoprotein complex of DNA, especially for the arrangement of the nucleosomes. Histones can undergo several modifications, but modification of a linker histone with a single ubiquitin (mono-ubiquitylation) remains one of the least understood epigenetic modifications. One reason is the inaccessibility of homogeneously modified linker histones for experimental methods, which are crucial for distinct studies. We combine molecular dynamics simulations with machine learning-based approaches to study the influence of mono-ubiquitylation in linker histones on DNA interaction and their ability to form higher order chromatin structures. We were able to determine the probable states in six differently linked histone-ubiquitin complexes via accelerating classical molecular dynamics simulations and using advanced state characterization techniques. While it is possible to access the chromatosome with ubiquitylated linker histone computationally, even faster sampling techniques like coarse-graining and Brownian dynamics can greatly benefit from an ensemble of atomistic structures. We developed an efficient geometric scoring technique to select biologically relevant structures of all six mono-ubiquitylated linker histones that can bind to nucleosome and present them as atomistic starting structures for further studies.

## Introduction

Chromatin is the nucleoprotein complex in cells in which DNA is packed. Besides the DNA it is composed of various histone and non-histone proteins. [1] The basic building block of chromatin is the nucleosome, a complex which is formed when ≈ 147–167bp of DNA wrap around an octameric protein complex of four pairs of core histones. [2] In chromatin, the nucleosome is accompanied by a linker histone and this complex is then called chromatosome. The linker histone interacts with the DNA linkers which connect the chromatosomes and thus directly influences higher packing of the chromatin [3], such as the formation and structure of the 30nm chromatin fiber. The nature of linker histone binding modes and chromatin packing is a topic of ongoing research. [4–10]

**Fig 1:**
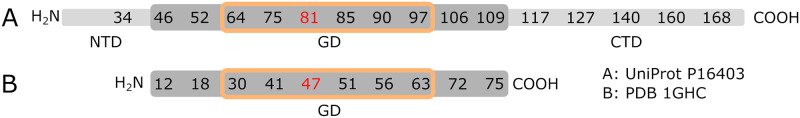
Schematic sequences of wild type human linker histone H1.2 (A) and the globular domain (dark grey) of chicken histone H1 (PDB 1GHC [38] B). The numbers represent aligned lysine residues that have been found to undergo ubiquitylation. [14–16] The red numbers indicate lysines considered in this study that have not been found to be ubiquitylated in nature (K47 in 1GHC and K81 in human H1.2). Lysines within the orange outline are investigated in this study. Abbreviations: NTD (N-terminal domain), GD (globular domain), CTD (C-terminal domain).

The binding affinity of the linker histone to the chromatin can be affected by the histone’s post-translational modifications, such as methylation, acetylation, phosphorylation, etc. [11] One such post-translational modification is the formation of an isopeptide bond between a substrate protein’s lysine residue and the C-terminus of the protein ubiquitin (Ub) (termed as “ubiquitylation” or “ubiquitination”). Ubiquitylation is known to occur at the core histones, where it can regulate, among others, gene silencing, [12] chromatosome dynamics, and ultimately DNA accessibility [13]. Later ubiquitylation was discovered to also occur on the linker histone (Fig 1). [14–16] Furthermore, polyubiquitylation of the linker histone has been identified as an intermediate in the repair of double strand DNA break. [17, 18] The chromatin system has also been studied in silico in various resolutions ranging from highly abstracted models where the nucleosome comprises a rigid body to multiscale and all-atom simulations of the nucleosome core structure. [19–23] However, most of the studies exclude the linker histone from their models, despite acknowledging the importance of this protein in the formation mechanism of higher order chromatin. The position of the linker histone in the “pocket” of linker and core DNA, i.e. the binding modes, has been subject of discussion but most probably varies between a symmetric on-dyad mode and an asymmetric off-dyad mode. [10, 24–27] Recently, all-atom and multiscale simulations have been conducted to further investigate the nucleosome/chromatosome system. [22, 23, 28–30] In general, simulation methods at all scales benefit from atomistic data. So has the nucleosome PDB ID 1KX5 been used for all-atom MD, coarse-grained MD, and Monte Carlo simulations. [31–35] While new efforts have been made to include the flexible tails of the linker histone, ubiquitylation of the linker histone remains underrepresented. [36]

First investigations assessing the influence of ubiquitylated linker histone (HUb) on the chromatosome complex suggested a trend towards a compaction of chromatosome arrays upon ubiquitylation. [37] However, some studies have shown that ubiquitylation of histones and especially K30 ubiquitylated linker histone (K64 in human H1.2, see Fig 1 for comparison of lysines’ numbering) prevent compaction, relax the structure, and promote transcription. [39, 40] Given the geometry of the system it seems astounding that Ub can form a covalent bond to the similar sized globular domain of the linker histone while still allowing a functional and ordered complex to form. Even more so, when an array of multiple chromatosomes is considered. [41] Structural data on ubiquitylated linker histones is still not available.

As many cellular processes depend on chromatin accessibility, static or dynamic structural data of HUb isolated and HUb inside a chromatosome would greatly benefit from further research. Structural data on the chromatosome is sparse: the protein database lists 8 such structures, as of this study: 4QLC [42] 5NL0 [37], 5WCU [43], 7DBP [44], 7KBF [45], 7K60, 7K5X, 7K5Y, 7K63 [46]. These chromatosomes mainly vary in the length of the linker DNA. All of them exhibit the on-dyad binding mode. We decided to use the three chromatosome structures obtained via X-ray crystallography as references (first three entries in Table 1). All of these structures are synthetic biological constructs reconstituted from core histones and the Widom 601 DNA sequence, which has been specifically tailored to increase binding to the core histones and thus has come under criticism. [47, 48] No structures let alone dynamic data of ubiquitylated linker histone have yet been published. Attempts to crystallize HUb in a chromatosome complex have not been fruitful so far. [49] In this study, we rely on the structural data summarized in Table 1 as basis for all further modeling. The structure in off-dyad binding mode obtained by molecular docking was kindly provided by Dr. Yawen Bai. [10] A notable example is the tetranucleosome array PDB 1ZBB crystallized by Schalch et al. [4] which we will use to bridge the gap between single nucleosome/chromatosome structures and higher order chromatin.

**Table 1:** Five chromatosome structures used as reference in this study: PDBs 4QLC, 5NL0, and 5WCU (rows 1–3) exhibit an on-dyad binding mode of the linker histone. The reference structure of the off-dyad binding mode (row 5) was obtained via HADDOCK docking using a nucleosome from 1ZBB and PDB 1HST as the histone. [10, 50]. PDB 1ZBB is used as a reference for a an array of multiple nucleosomes. The last row represents the present study. We use PDB 1GHC as the linker histone (also see Fig 1 for a sequence alignment of the linker histones).

Method	PDB ID	binding mode	Year	Core histone organism	Linker histone organism	DNA sequence	Reference
X-ray	4QLC	on-dyad	2014	*D. melanogaster*	*G. gallus*	Widom 601	Zhou et al. [42]
X-ray	5NL0	on-dyad	2017	*X. laevis*	*X. laevis*	Widom 601	Bednar et al. [37]
X-ray	5WCU	on-dyad	2018	*D. melanogaster*	*G. gallus*	Widom 601	Zhou et al. [43]
HADDOCK	-	off-dyad	2013	*X. laevis* (taken from 1ZBB)	*G. gallus* H5 (PDB 1HST)	Widom 601	Zhou et al. [10]
X-ray	1ZZB	-	2005	*X. laevis*	-	Widom 601	Schalch et al. [4]
**molecular modeling**	**all**	**both**	**2023**	**all**	***G. gallus*** **H1 (PDB 1GHC)**	**Widom 601**	**this study**

**Fig 2:**
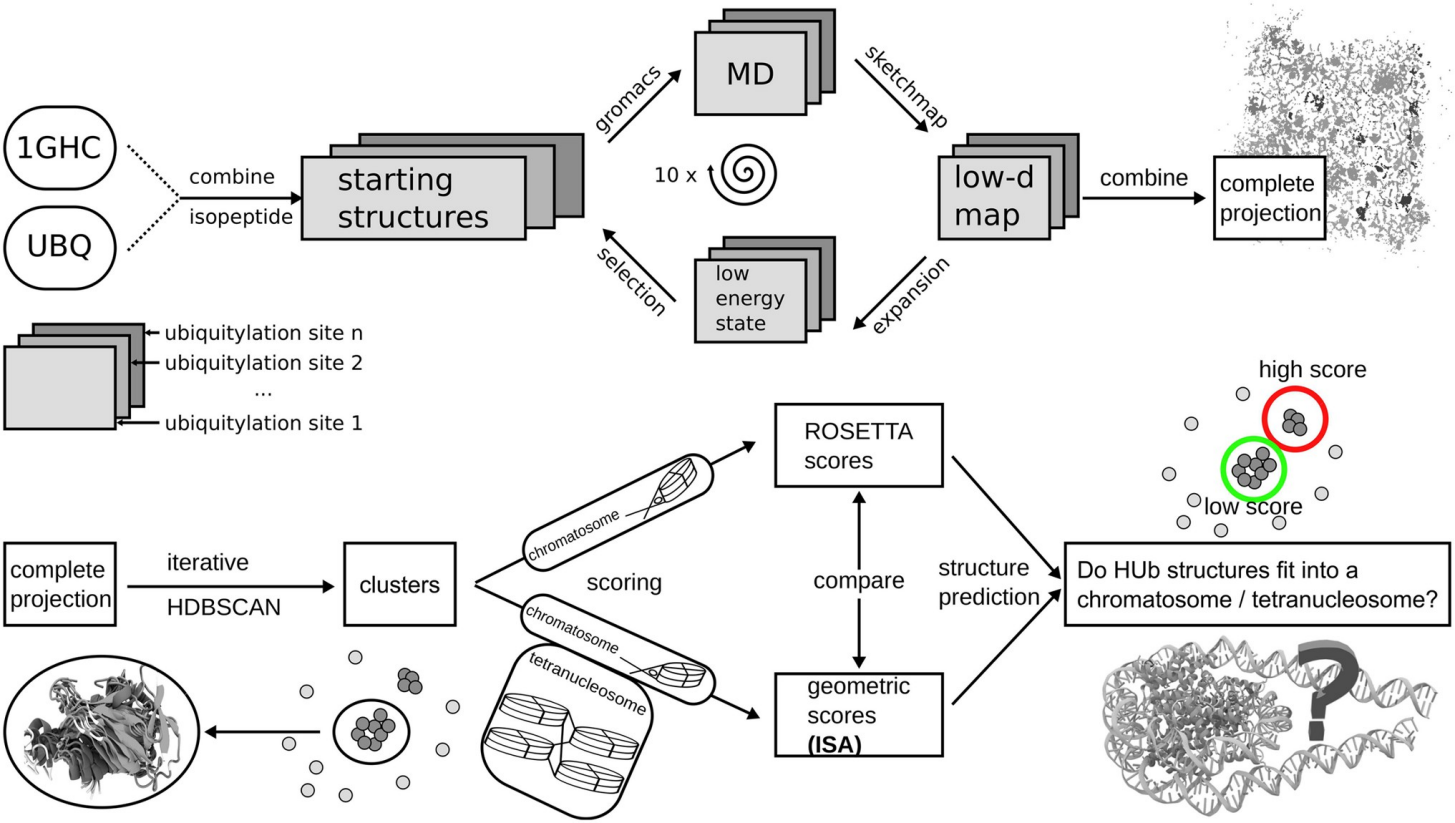
Illustration of data-generation (top half) and analysis (lower half) workflow to identify possible structures of ubiquitylated linker histone in a chromatosome. Data was produced by MD simulations driven by an expansion scheme which selects new starting structures from sparsely populated regions of phase space. The aggregated MD data is projected into a combined low-dimensional space and clustered iteratively. Clusters (i.e. characteristic conformational states) are then fitted into different chromatosome structures using a new geometric scoring method.

The X-ray and HADDOCK methods provide valuable first steps for structural data on the chromatosome. However, they strive to find a single structure of an inherently dynamic system. In this paper we aim for the combination of molecular dynamics simulations with machine learning techniques to explore the conformational landscape of ubiquitylated linker histone. We offer possible conformations of HUb in chromatosomes and chromatosome arrays to reveal a novel view into DNA regulation by ubiquitylation. We generate an extensive library of conformations of differently ubiquitylated linker histones using molecular dynamics (MD) simulations guided by our own enhanced sampling algorithm [51]. We reduce the data size by excluding statistically insignificant/physically improbable structures through clustering in a dimensionally-reduced space. We present a particularly efficient geometric scoring method for this system which is then used to analyze if and how the ubiquitylated linker histone fits into the chromatosome—and if this is dependent on the ubiquitination site. Last but not least we use the same methods on a tetranucleosome array to bridge the gap between isolated chromatosomes and chromatin fibers. The workflow employed in this study is visualized in Fig 2. It can be summarized into three main steps: 1) exploration of conformational phase space; 2) identification of representative conformational states; 3) scoring of HUb in on-dyad and off-dyad mono chromatosomes, and a tetranucleosome array.

## Results

We make general assessments of the conformational properties of the six HUb variants and propose the identified clusters as structurally stable sub-states to be used as input for the interpenetration and scoring algorithm presented in Materials and methods. Doing so, we are able to screen through all simulation frames and find HUb conformations that fit into the DNA “pocket” of a nucleosome and a tetranucleosome array.

### Structural diversity of six differently ubiquitylated histones

**Fig 3:**
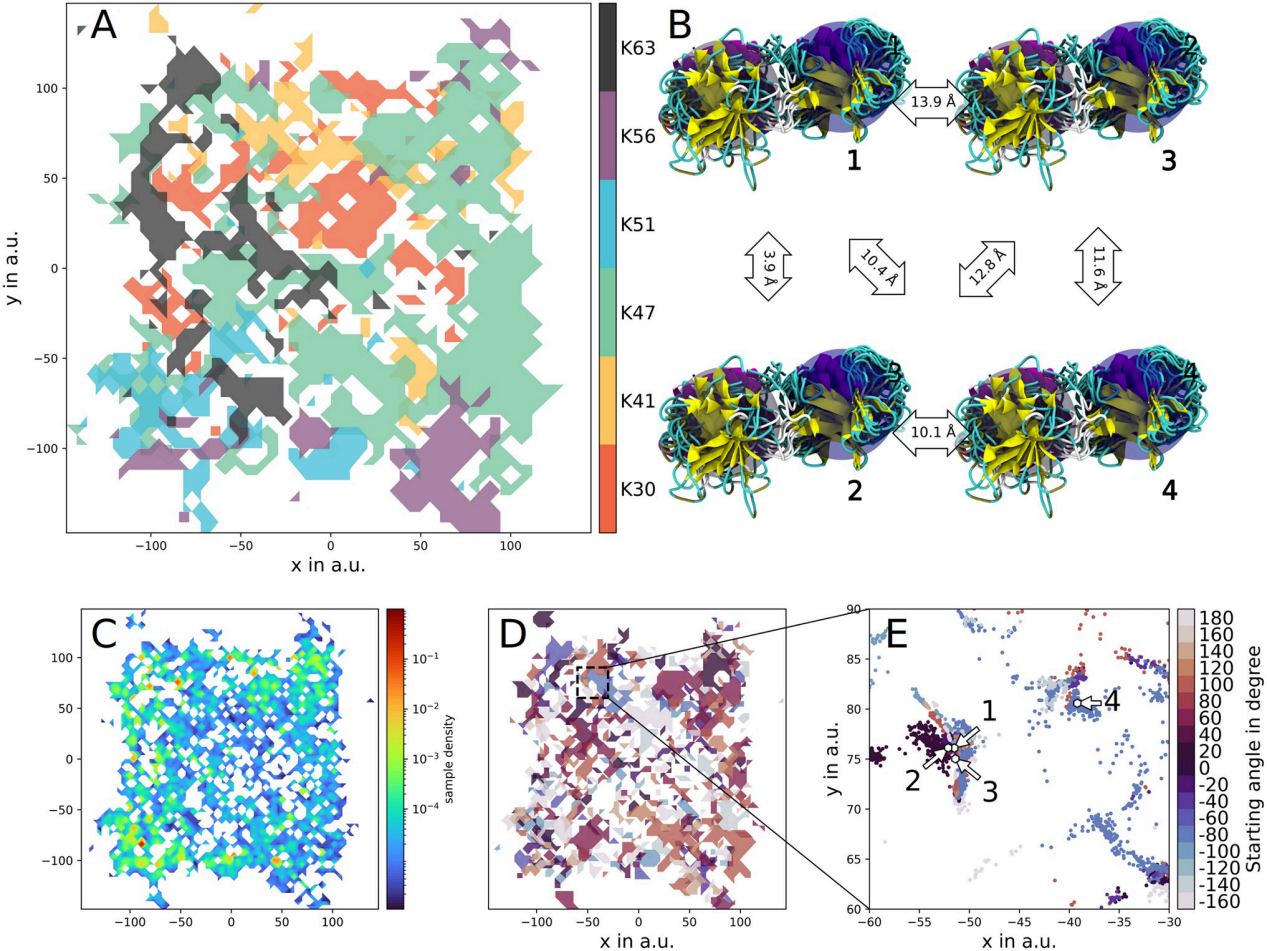
Sketch-map projections of all conducted simulations colored by various parameters and exemplary structures. A contour plot with transparent patches colored according to ubiquitylation site (A) shows that the most distinct feature of the six HUb variants is the ubiquitylation site. A density map of the combined projections (C) shows that the low-dimensional representation is mostly shallow (blue) with only small regions of higher density (red). Coloring the combined projection according to the starting χ_3_ angles ((D) and (E)) shows a finer structure of the projection. Four exemplary structures from (E) have been chosen and their RMSD centroids are visualized with cartoon representation and colored according to secondary structure features (B). The blue transparent sphere is focused on the histone subunit, the grey one on the Ub subunit. Closeness in sketch-map space is loosely related to structural similarity, due to sketch-map’s non-linear dimensionality reduction. In (A) regions of high density and high RMSD variance were excluded, to not bias the density towards these regions. Subfigure (C) was rendered after removing the high-density, high-RMSD variance region (Sec. S2.3, S2 Text, S5 Fig).

The complete set of 1180 simulations (≈ 35 μs of aggregated simulated time) of the six HUb variants was projected into the same low-dimensional space with the sketch-map dimensionality reduction algorithm. [52] In this projection the closeness of any two points is related to the structural similarity of the respective protein’s conformations. Patches with high density contain similar structures of very little Root mean square deviation (RMSD) variance, whereas solitary points represent statistically insignificant structures which can be—especially after the exhaustive sampling conducted here—viewed as transition states. RMSDs between structures belonging to high-density basins are loosely proportional to their respective distance in sketch-map space, however, they are not linearly related (Fig 3B and 3E). Using this method we could identify large regions of unique structures for every ubiquitylation variant and small intersections of these regions, where the conformational space of two or more variants overlap (Fig 3A). The starting sidechain dihedral angle χ_3_ is the second most prominent feature (Fig 3D). These map regions cannot be taken as statistical weights for certain conformations as for that a notion of density is required (Fig 3C). After the removal of high density regions (Sec. S2.3, S2 Text, S5 Fig) the density can be compared with the similar di-Ubiquitin protein (diUb). The density map is more shallow than similar diUbs. [53] This is due to the histone’s conformational space which is greater, when compared to ubiquitin (i.e. the histone is more flexible) and the expansion scheme driving the simulation away from meta-stable, local-minima conformations. We additionally questioned the completeness of the conducted sampling and stability of obtained meta-stable states, but assessing ergodicity is out of the scope of this work.

After creating the exhaustive structural library for all six HUb variants we can address more interesting questions on: how many meta-stable states does HUb assume and which of these states can bind to a nucleosome.

### Identification of characteristic conformational states of HUb

The visual inspection of the 2D sketch-map projections already reveals the enormity of the conformational landscape of the six variants. Each point in sketch-map space represents one conformation sampled by an unbiased MD simulation. High density regions represent statistically significant sub-states of the variant’s combined phase space. The task of identifying these states can be reduced to a grouping of similar features—clustering.

Considering size and properties of the given data there are some requirements to a clustering method to be able to define meta-stable states in the 2D sketch-map projection: 1) handle vast amount of points; 2) work without requiring a predefined number of clusters; 3) distinguish between dense basins of any size and shape (meta-stable configurations) and low density background (transition states).

We found the hierarchical density-based clustering algorithm HDBSCAN with iterative application particularly well suited for this task. [54] HDBSCAN transforms the space before building a single linkage tree thus pushing “noise” (sparse regions) further away from the dense data. We applied this algorithm iteratively, first to exclude high density regions with high RMSD variance (artifacts of the projection algorithm, see Sec. S2.3, S2 Text, S5 Fig), and then to gradually define clusters proceeding from large to small by decrementing the hyperparameter (more details on the algorithm can be found in Materials and methods: Identifying characteristic conformational states of HUb).

**Fig 4:**
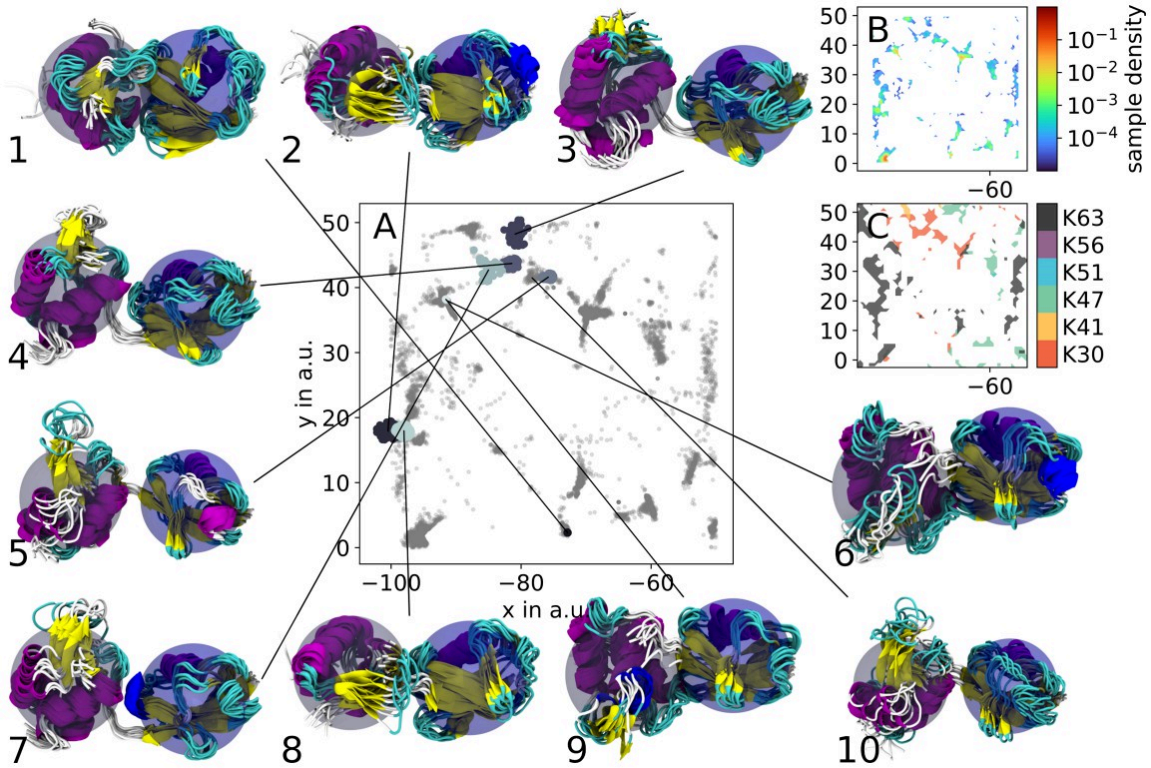
Applying an iterative HDBSCAN clustering, a total of 496 clusters were obtained from the complete sketch-map projection. A selection of a few clusters from a section of the joint projection (compare Fig 3A, 3C and 3D for overview) is visualized as colored patches, whereas the outliers are grey and slightly transparent (A). Structure renders of these clusters (blue sphere centered on H1 subunit, grey sphere centered on Ub subunit) show that a satisfying degree of structural cohesion could be achieved. Subfigures (B) and (C) show the sample density and the dominant ubiquitylation site of the region in (A), respectively.

With this iterative clustering approach we extracted 496 clusters which represent ≈ 30% of the variants’ conformational space (Fig 4). Compared to just observing the 2D projections of the phase spaces of differently linked HUb, clustering allowed us to better analyze the joint phase space of HUb’s. Out of the 496 clusters only 64 (12% of all clustered points, 2% of all points) were sampled by a single trajectory. This points towards a convergence of the conformational space sampled by the MD simulations. A fifth of all clustered points (keep in mind that clusters can have different populations) are part of clusters that contain structures from multiple topological different variants. This implies meta-stable HUb states, which are not specific to the linker position, and this can be a hint to the question, why no significant difference can be seen in the experiments for different ubiquitylation sites. [49] The presence of clusters with differently ubiquitylated variants also confirms the advantage of using our expansion scheme compared to straightforward MD, as applying sketch-map and HDBSCAN to the initial simulations (S2 Fig) resulted in clusters exclusively built from single trajectories.

### Scoring HUb conformations into nucleosome structures

#### Scoring single structures into nucleosomes

**Fig 5:**
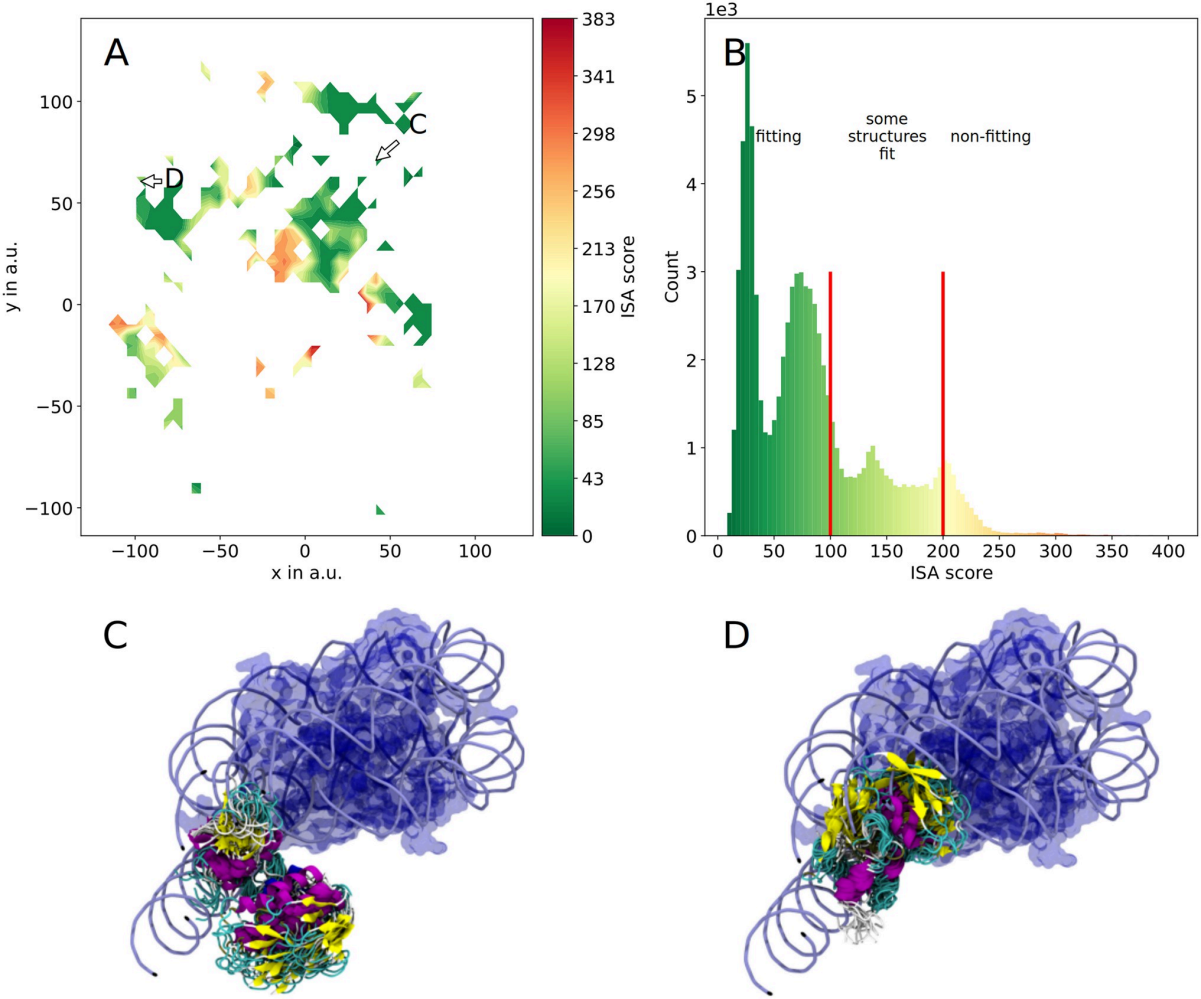
Results of the interpenetration and scoring algorithm and example structures for scoring K30HUb into 5NL0. The sketch-map projection (A) colored according to ISA score exhibits distinct regions with fitting (green, low score) and non-fitting (red, high score) conformations. The histogram of scores has a skew to lower scores (B). In (C) and (D) exemplary HUb clusters are rendered within the nucleosome of 5NL0, their location in sketch-space is annotated in (A). Both (C) and (D) visualize the DNA as tubes and the core histones as a transparent blue surface. The HUb structure bundles are rendered using their secondary structure and colored accordingly. In (C) the K30HUb cluster with the lowest ISA score is shown. Using this cluster the HUb chromatosome complex could easily be used as input for further simulations. In (D) a cluster with an ISA score of 150 is shown. This cluster can be taken as an example of a high-score cluster, that does only slightly intersects with the DNA linkers.

The interpenetration and scoring algorithm (ISA) allows a fast scoring of all simulated HUb conformations (see Materials and methods: Fitting HUb into the chromatosome). As a first introduction to the ISA scores we will use K30Ub and place it into the 5NL0 nucleosome. The sketch-map projection can now be colored by the score, revealing distinct regions of higher and lower scores (Fig 5A). The lowest ISA score observed for placing K30Ub into 5NL0 is 8.4 and the highest is 406.2. The previously identified clusters represent (meta-)stable sub-states on the protein’s folding pathway and as such are good candidates to identify states of lower scores which are also likely to occur in real-life systems.

**Fig 6:**
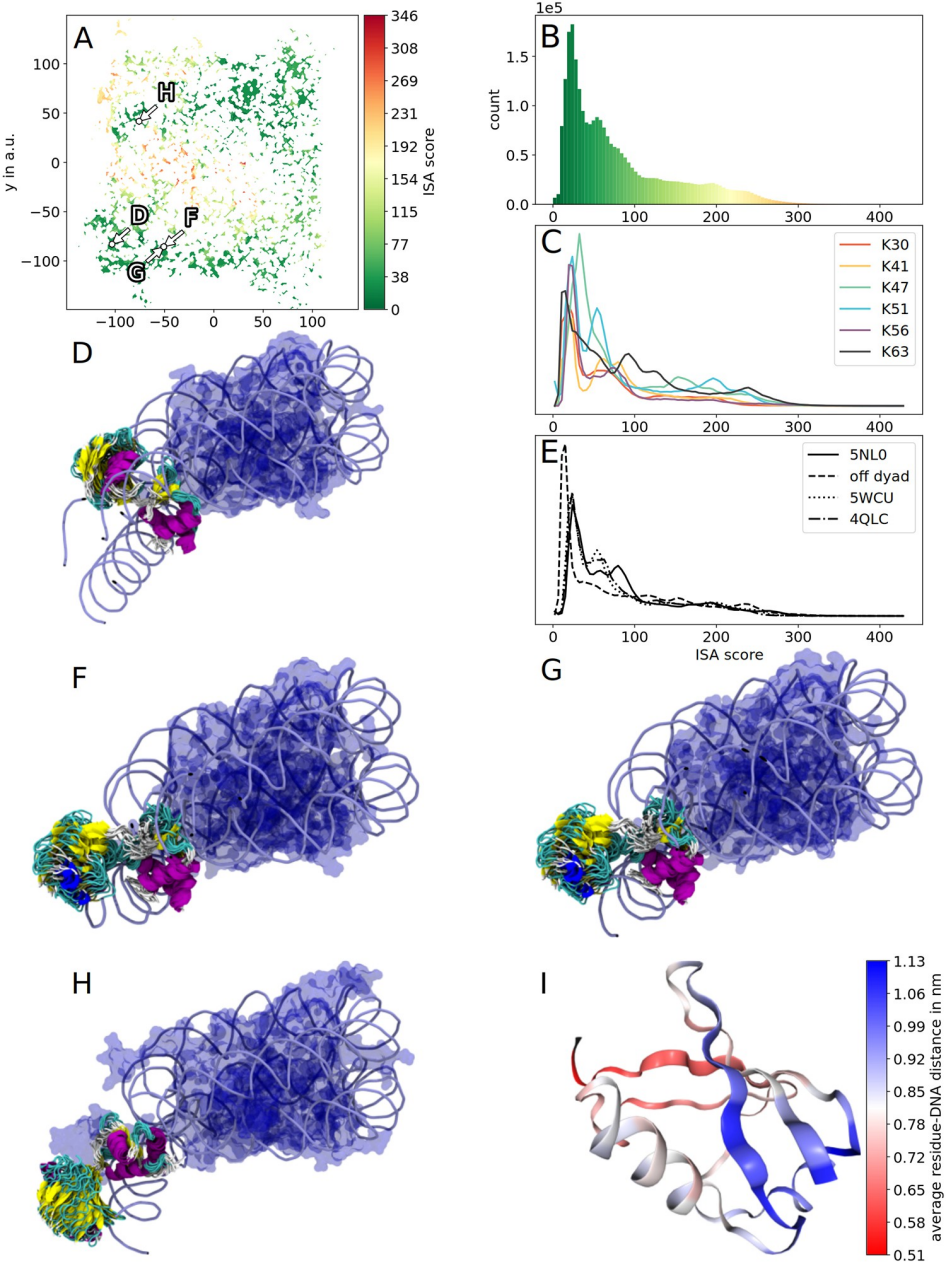
Scores and renders of the six HUb variants in the four chromatosome structures. In (A) the sketch-map projection is colored according to the ISA score where a specific region exhibits high scores and contains non-fitting HUb conformations. The distribution of scores can be seen in (B), (C), and (E). The clusters with the lowest scores of the parent chromatosomes 5NL0, 5WCU, 4QLC, and the off-dyad structure are shown in (D, F-H), their location annotated in (A). Coloration of renders is in line with Fig 5. Note, that the lowest scoring cluster for 5WCU (F) and 4QLC (G) is the same cluster. Both chromatosomes are structurally very similar, missing the DNA linkers that are present in 5NL0. Also note, how the Ub subunit of the lowest scoring cluster fitted into the off-dyad chromatosome points into a different direction. The off-dyad structure in (H) contains the tail domains of the core histones. In (I), the crystal structure of 1UBQ was colored according to the average per-residue minimal distance to DNA after placing HUb into 5NL0. The possible values range from red (closest) to blue (farthest).

We used 4 different chromatosome structures as receptors (rows 1–3 on-dyad H1 binding mode, row 4 off-dyad binding mode in Table 1). Thus, the resulting scores can not only be split up into the different ubiquitylation sites (Fig 6C) but also into the different receptor nucleosomes Fig 6E). See S8 Fig for the comparison of all ISA scores for monochromatosomes. Although the number of atoms varies for these nucleosome structures we still can assume that low and high scores can be compared within the margin of error. A score of 0 is achievable when no intersections occurs at all. ISA scores ranging from 0 to 473 were observed. We chose an ISA score of 100 as a cutoff (the 75th percentile of all scores lies at 89.9, which after visual inspection of edge cases was deemed a good choice (S9 Fig)) and conformations with scores lower than 100 were designated as “fitting”. Structures in between scores of 100 and 150 typically intersect with the DNA linkers (e.g. Fig 5D) or the nucleosomal core. If an intersection with the DNA linker occurs the structure could potentially still be used for MD simulations after energy minimization, which is not pursued here. Protein conformations with scores of 150 and above are designated as not fitting.

The distribution of scores is skewed to lower scores. Although the HUb proteins were simulated in water without any nucleic acid present, the overall majority of sampled structures fits into the nucleosome (Fig 5B). This coincides with studies observing reconstituted HUb chromatosomes to still form orderly complexes and not break the complex completely. [39] The chromatosome with off-dyad binding linker histone exhibits greater deviations because its core histones contain the flexible tails, that the other chromatosomes lack. The superposed conformations of the ubiquitylated linker histones can overlap with these regions. However, due to the flexibility of these tails [55] we expect the tails to be able to accommodate for the ubiquitin subunit and possibly interact with it in a dynamic system.

#### Scoring clusters into nucleosomes

When fitting clusters (i.e. bundles of 100 to 1000 HUb conformations) into the chromatosomes, the ubiquitin subunit does often fan out. Despite this structural variance and the entailing increase in the conformational demand of the Ub subunits, the majority of the obtained clusters did fit surprisingly well into the chromatosomes. All clusters do exhibit a non-zero mean ISA score, with the best fitting cluster (Fig 5C) having a mean ISA score of 18 (see S1 Table for the overview of the 50 largest clusters). Using the four chromatosomes from Table 1 we found that for 4QLC and 5WCU the minimal ISA score is achieved by the same cluster (Fig 6). Interestingly, the HUb structure bundles of all on-dyad chromatosomes exhibit similar spatial positioning. Here, the Ub subunit of the lowest scoring cluster is situated “above” the nucleosomal core. In contrast, in the off-dyad nucleosome the DNA linkers exit the nucleosomal core at other angles than in the on-dyad nucleosomes and the linker histone in the off-dyad nucleosome is slightly shifted and rotated. This leads to the lowest scoring clusters for the off-dyad nucleosome being distinctively different from the on-dyad nucleosomes. Here, the Ub subunit points outward. Using the scores of clusters the question whether ubiquitylated linker histone can fit into the chromatosome could be answered. As most of the clusters exhibit a lower score we assume that HUb has a tendency to assume conformations that fit into the DNA pocket of these mono-nucleosomes. However, biological systems of chromatin are comprised of many nucleosomal building blocks. Thus, investigating the positioning of HUb in an array of nucleosomes is crucial to gain deeper insights into these systems.

#### Tetranucleosome array

**Fig 7:**
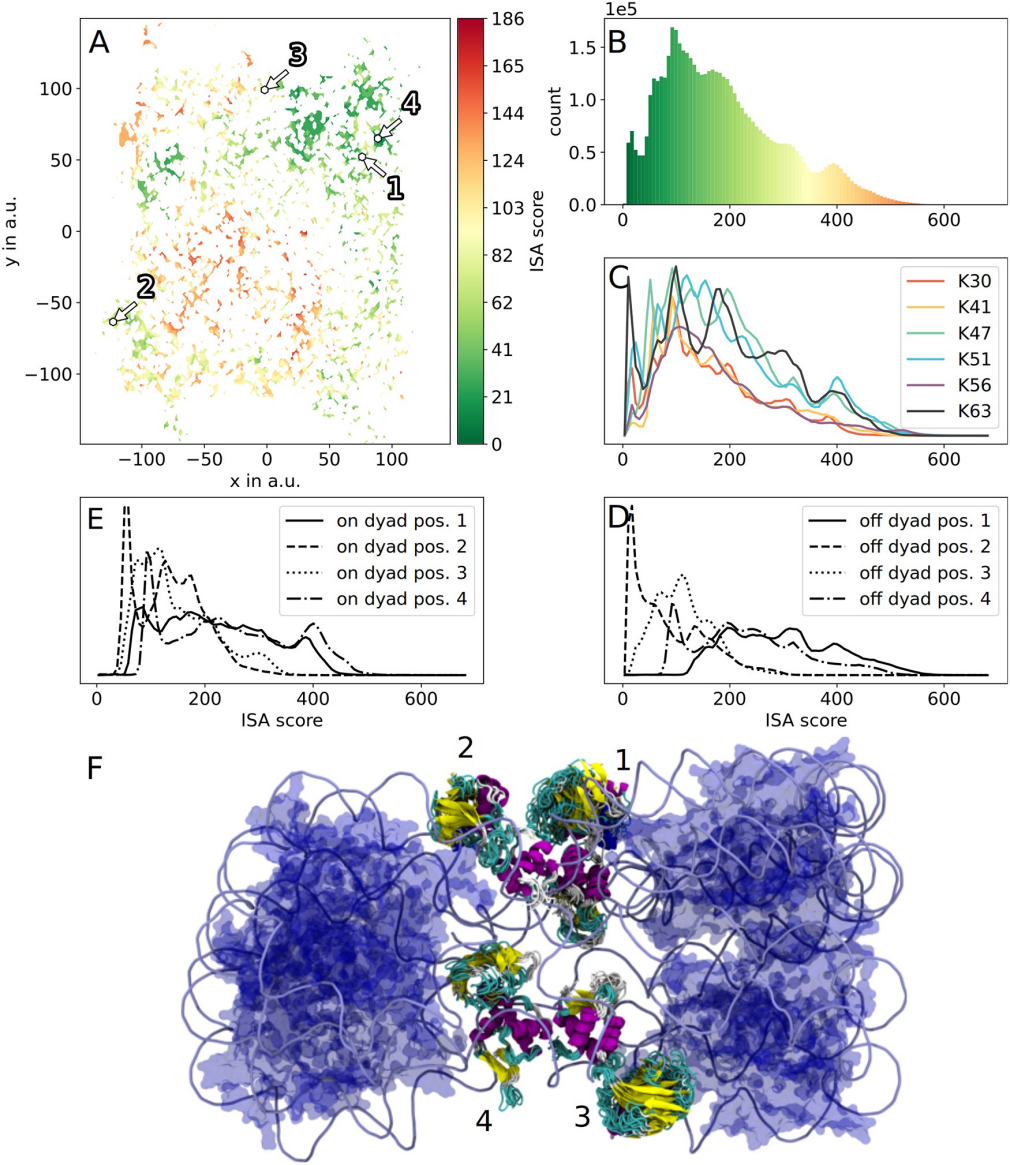
Using ISA to gauge the positions of HUb in a tetranucleosome array. The median ISA score over the 8 possible HUb positions (4 nucleosomes in the array using on-dyad and off-dyad binding motifs) exhibit large regions of higher ISA scores (A). In (B-E) the distribution of ISA scores is visualized for all 8 positions combined (B), differentiated by ubiquitylation site (C), and divided by the 4 off-dyad and 4 on-dyad HUb positions (E) and (D), respectively. The render in (F) uses the on-dyad 5NL0 chromatosome as reference structure for the linker histone positions 1 and 4 and the off-dyad reference structure for the positions 2 and 3. The geometric cluster centroids are annotated in (A) accordingly. They exhibit mean ISA scores between 12 and 86. These clusters are chosen for illustrative purposes to exhibit how certain sub-states in the conformational landscape of all HUb variants can fit into the more restrictive tetranucleosome array. For the four positions 1, 2, 3, and 4, the chosen clusters contain predominantly the linkage types K51, K30, K51, and K41, respectively. The DNA is rendered as light blue tubes, core histones are blue translucent. Secondary structure elements are colored as default in VMD. [56]

As a final step to find suitable conformations of HUb inside chromatosomes we also applied our procedure to a tetranucleosome array published by Schalch et al. (PDB 1ZZB). [4] Similar to most chromatin complexes on the protein database, this X-ray structure omits the linker histone. We approximated theoretical positions of the 4 linker histones in this complex by protein sequence and position alignment of the core histones in the tetranucleosome array with PDB 5NL0 and the off-dyad chromatosome (Materials and methods: Fitting HUb into the chromatosome and S3 Table). A position histogram from the atomic positions of the tetranucleosome was created to thereupon construct the scoring histogram in the same fashion as for the other nucleosomes. Subsequently, all HUb conformations were again put into each of the 4 possible linker histone positions and the corresponding ISA score was calculated Fig 7). The same regions that previously resulted in higher scores now also result in high scores for the tetranucleosome array. However, the high score region is more extended and only the conformations located in the upper right corner of the sketch-map projection should be considered as “fitting” with a small region of fitting structures around -100, -50. The “per-histone-position score distributions” (B) and (C) are created for the 4 on-dyad and off-dyad positions, respectively. The cluster with the lowest score is selected for each of the on-dyad positions and rendered in (D). The cluster centroids are marked accordingly in (A). Clusters located in positions 2 and 3 generally exhibit lower scores than 1 and 4. At this point it is not clear whether this is due to the limited length of the tetranucleosome array or whether longer nucleosome arrays exhibit more similar per-histone-position scores over their full length. To model such a system more research needs to be conducted. The packaging (on which the scores depend) of the nucleosomes is still a topic of ongoing discussion. However, our findings strongly suggest that there are native HUb conformations that fit well into the limited space available in a nucleosome array (see S2 Table for the composition and scores of the ubiquitylation sites).

Additionally to the ISA scores for the HUb variants, we looked at the position of the ubiquitin with respect to DNA. For this, all sampled conformations of the six variants were placed into 5NL0. The mean per-residue distance from ubiquitin to DNA is used to color the crystal structure 1UBQ (Fig 6I). The α_1_ helix of the Ub-subunit exhibits shorter distances (white) to the DNA than most of the residues in the β-grasp region (blue).

## Discussion

In this work we have shown that differently ubiquitylated avian linker histones take on a wide range of conformations that are mainly dictated by the chosen ubiquitylation site although some intermixing of regions in phase space can be observed. MD simulations in combination with an expansion scheme that accelerates the exploration of phase space created a large and exhaustive library of HUb conformations. The scheme explores the relatively shallow free-energy landscape with many conformational sub-states that were identified by density based clustering on two-dimensional representation of the conformational space. These clusters represent local minima and were visited by multiple simulations from the same, sometimes even from different, topologies. The clusters pose as statistically significant (meta)stable sub-states that a real-life HUb protein might adapt. Compared to other structural methods like NMR spectroscopy or X-ray crystallography we created dynamic data of a protein modeling its time-resolved conformational changes.

We developed an interpenetration and scoring algorithm (ISA) that exploits the geometric properties of the system and shows that complex and computationally expensive scoring functions are not needed if the systems are large enough. A good correlation with pyROSETTA could be achieved by implementing cumulative 3D histograms of atomistic positions. With that we were able to identify regions of the combined HUb phase space that contain clusters that fit well into any of the 4 available chromatosome structures. Although the nucleosomes were not present during our simulations, we found roughly 93% of all conformations to be fitting into the DNA pocket of the nucleosome. This strongly indicates that the HUb protein has a natural affinity to be placed in this environment. The HUb conformations are well suited for modeling HUb inside a nucleosome and could easily be taken as input structures for further investigation via MD simulations as they generally do not need energy minimization. Such simulations don’t need to be long to probe the interactions between the Ub subunit and the DNA or the core histones. Simulations at higher scales can also benefit from these input structures and investigate the dynamics of HUb in the chromatosome system on longer timescales. These techniques can be especially beneficial to probe interactions with the post-translational-rich and disordered tails of the histones, which also interact with the DNA and could compete with Ub. [57–60] Moreover, the ensemble obtained in our study will be affected/shifted by changes in the environment, the further interaction partner (and the tails). Nevertheless, these fitted structures are extremely valuable for subsequent simulation studies. Furthermore, higher order HUb chromatin can be made accessible with these simulation techniques. Our analysis of the 1ZBB tetranucleosome has shown that, even though the DNA-pockets of the tetranucleosome array are more restrictive, multiple clusters could easily be placed into it without significant clashes, independent whether an on-dyad or off-dyad chromatosome was used to approximate the linker histone positions.

Additionally, we found that the ubiquitin subunit in our simulations has the α_1_ -helix (beside the isopeptide bound N-terminal region) closer to the DNA than the β-grasp of Ub (Fig 6I). Whether the 3 lysine residues in the α_1_ -helix could favorably interact with the DNA is certainly a worthwhile question for further examinations of this system.

Our insights into how ubiquitylated linker histone might fit into chromatosomes can help in further developing models for the stabilization of chromatin.

## Materials and methods

### Enhanced Sampling of HUb Conformations

We created starting structures for MD simulations by linking the globular domain of avian linker histone H1 (PDB 1GHC [38]) and ubiquitin (PDB 1UBQ [61]) via an isopeptide bond between H1’s K30, K41, K47, K51, K56, and K63, residues and ubiquitin’s C-terminal glycine using the program UCSF Chimera [62] (matching lysine indices in sequence aligned human linker histone H1.2 are displayed in Fig 1). These proteins will be called K30Ub, K41Ub, K47Ub, K51Ub, K56Ub, and K63Ub, respectively. Simulations were performed for 1 to 3 different initial rotations of the lysine’s sidechain dihedral χ_3_ angle for ≈ 1 μs using GROMACS program package [63] with the GROMOS54a7 forcefield [64] (for more detailed information about simulation parameters and protocol refer to Sec. S1.2 in S1 Text). After already ≈ 100 ns of the 1 μs simulated time the simulations of the different variants collapsed into compact distinct HUb structures, which exhibited little structural changes for the remainder of the simulations (S1 Fig). The long simulations could not decorrelate from the influence of the rotation angle χ_3_ in the starting structure even in 1 μs simulation time and became “stuck” with little variations in conformation.

To reduce bias of the starting rotation of the χ_3_ angle and push the systems to fully explore their conformational space with all-atom MD without adding biasing potentials we chose to start more rotamers of the variants by additionally varying the χ_3_ sidechain dihedral angle in 20° steps. All in all 6 × 18 = 108 starting structures were created. As the exploratory long simulations had collapsed quite rapidly, we decided on 20 ns long initial simulations from which then an expansion scheme was started that is tailored to push the system to explore new regions of conformational phase space instead of getting stuck in few collapsed structures.

This expansion scheme has already been successfully used to study the conformational space of intrinsically disordered peptides. [51] It accelerates the sampling by running many parallel simulations and assessing in regular intervals whether simulations have been trapped in low-energy basins. Identification of these basins is done by binning a low-dimensional projection of the conformational space of the HUb variants. For this reduction in dimensionality, we first needed to obtain high-dimensional descriptors of HUb that represent the protein’s conformation. For this system we chose to use the distances between the Cα -atoms to provide these high-dimensional descriptors, henceforth called collective variables (CVs). A conformation of the HUb can then be represented as a collection of these distances. Instead of using the full set of (1512)=11325 pairwise distances we chose to compare two different subsets of CVs for the expansion scheme: the solvent accessible surface area (SASA)-CVs and the residue-wise minimal distance collective variables (RMD-CVs). SASA-CVs were calculated according to Eq (1) by only using the Cα atoms with a SASA greater than 1 in the respective crystal structures. RMD-CVs have been successfully employed to characterize di-Ubiquitin. [65] These CVs were obtained by calculating the row-wise minimum of the pairwise distance matrix between the Cα atoms of the histone and ubiquitin subunit according to Eq (2).

$$\begin{array}{l} D^{304}=\text{vec}\left({\text{SASA}\_\text{dist}}^{16\times 19}\right)=\\ \text{vec}=\left(\begin{array}{cc} {\text{SASA}\;\text{C}}_{\alpha }\;\text{of}\;\text{H1}\;\text{subunit} & \overset{{\text{SASA}\;\text{C}}_{\alpha }\;\text{of}\;\text{Ub}\;\text{subunit}}{\overset{︷}{\left\{\left[\begin{array}{cccc} d_{1,11} & d_{1,16} & \cdots & d_{1,73}\\ d_{8,11} & d_{8,16} & \cdots & d_{8,73}\\ \vdots & \vdots & \ddots & \vdots \\ d_{75,11} & d_{75,16} & \cdots & d_{75,73} \end{array}\right]\right.}} \end{array}\right)=\left[\begin{array}{c} d_{1,11}\\ d_{1,16}\\ \vdots \\ d_{75,73} \end{array}\right] \end{array}$$ (1)

$$\begin{array}{l} D^{75}={\text{min}\_\text{dist}}^{1\times 75}=\\ =\begin{array}{cc} {\text{All}\;\text{C}}_{\alpha }\;\text{of}\;\text{H1}\;\text{subunit} & \overset{{\text{All}\;\text{C}}_{\alpha }\;\text{of}\;\text{Ub}\;\text{subunit}}{\overset{︷}{\left\{\left[\begin{array}{c} \min\;\left\{d_{1,1},d_{1,2},\cdots d_{1,76}\right\}\\ \min\;\left\{d_{2,1},d_{2,2}\cdots d_{2,76}\right\}\\ \vdots \\ \min\;\left\{d_{75,1},d_{75,2},\cdots d_{75,76}\right\} \end{array}\right]\right.}} \end{array}=\left[\begin{array}{c} \min\;d_{1}\\ \min\;d_{2}\\ \vdots \\ \min\;d_{75} \end{array}\right] \end{array}$$ (2)

Using either of the CV datasets (K47Ub, K41Hub, K51Ub, and K63HUb with SASA-CVs, K30Ub and K56Ub with RMD-CVs) we were able to project the 18 × 20 simulations into a two-dimensional projection (sometimes also called map) using the sketch-map [52] method (S2 and S3 Figs). In these projections every conformation is represented by a point; each point can be assigned to one specific structure in the conducted simulations. Note that for driving the expansion scheme, separate 2-dimensional projections of the six variants were generated (only later for analysis purposes the different systems were projected together into a combined low-dimensional map). After every 20 ns the simulations were stopped, re-projected and the expansion scheme was applied to yield 20 new starting structures. After the 3rd expansion step new landmarks were selected and the expansion scheme was continued for 6 more times (1 initial simulation run, 9 expansions) resulting in a total of ≈35 μs simulated time (Eq (3)).

$$\begin{array}{cc} T_{\text{total}} & \approx n_{\text{variants}}\times n_{\text{starting}\;\text{simulations}}\times T_{\text{starting}\;\text{simulation}}\\ & +N_{\text{expansions}}\times (n_{\text{variants}}\times n_{\text{continuing}\;\text{simulations}}\times T_{\text{expansion}\;\text{simulation}})\\ & +T_{\text{long}\;\text{simulations}}\\ T_{\text{total}} & \approx 6\times 18\times 20\;\text{ns}+9\times (6\times 20\times 20\;\text{ns})+12\;\mu \text{s}=35\;\mu \text{s} \end{array}$$ (3)

### Identifying characteristic conformational states of HUb

Subsequently all 483 983 conformations from the ≈ 35 μs aggregated simulation time were projected into a combined 2-dimensional map using the SASA-CVs to allow for analyses to work on all ubiquitination variants. A joint projection was a key argument in describing and comparing different Ub dimers by Berg et al. [65] But contrary to the projection of Ub dimers which used RMD-CVs, we found the SASA-CVs to be more suitable for a combined projection (sec. S2.2 in S2 Text, S4 Fig). From this projection, conformational states were determined with the help of a density based clustering method, HDBSCAN. [54]

HDBSCAN was first used with a minimal cluster size of 1000 to exclude projection artifacts (Sec. S2.3, S2 Text, S5 Fig). After the exclusion of these artifacts further clustering in the 2-dimensional space was carried out iteratively to obtain structurally consistent clusters. HDBSCAN takes at least one hyperparameter for its clustering algorithm. This parameter defines the minimum cluster size to differentiate between clusters and noise points after HDBSCAN’s space transformation. We reduced the hyperparameter step-wise from large to small clusters as not every sub-state of the protein’s phase space exhibited a similar density. The minimal cluster size was set to 750, 500, 250, 125, 75, 50, and 25, respectively, in 8 clustering passes. After each pass the homogeneity of structures in the obtained clusters was checked as follows. First, a cluster’s RMSD centroid was determined by using the python package MDTraj [66]. Second, the RMSD between the centroid and the remaining cluster points was calculated. The same was done for the distance in the two-dimensional projection. Third, a set of criteria were checked:

1. A mean RMSD distance between the cluster’s RMSD centroid and the other structures greater than 6 Å.
2. A Fisher-Pearson coefficient of skewness of the sketch-space distance distribution greater than 0.5.
3. Multimodality of the distribution as calculated by the python package unidip. [67]

**Fig 8:**
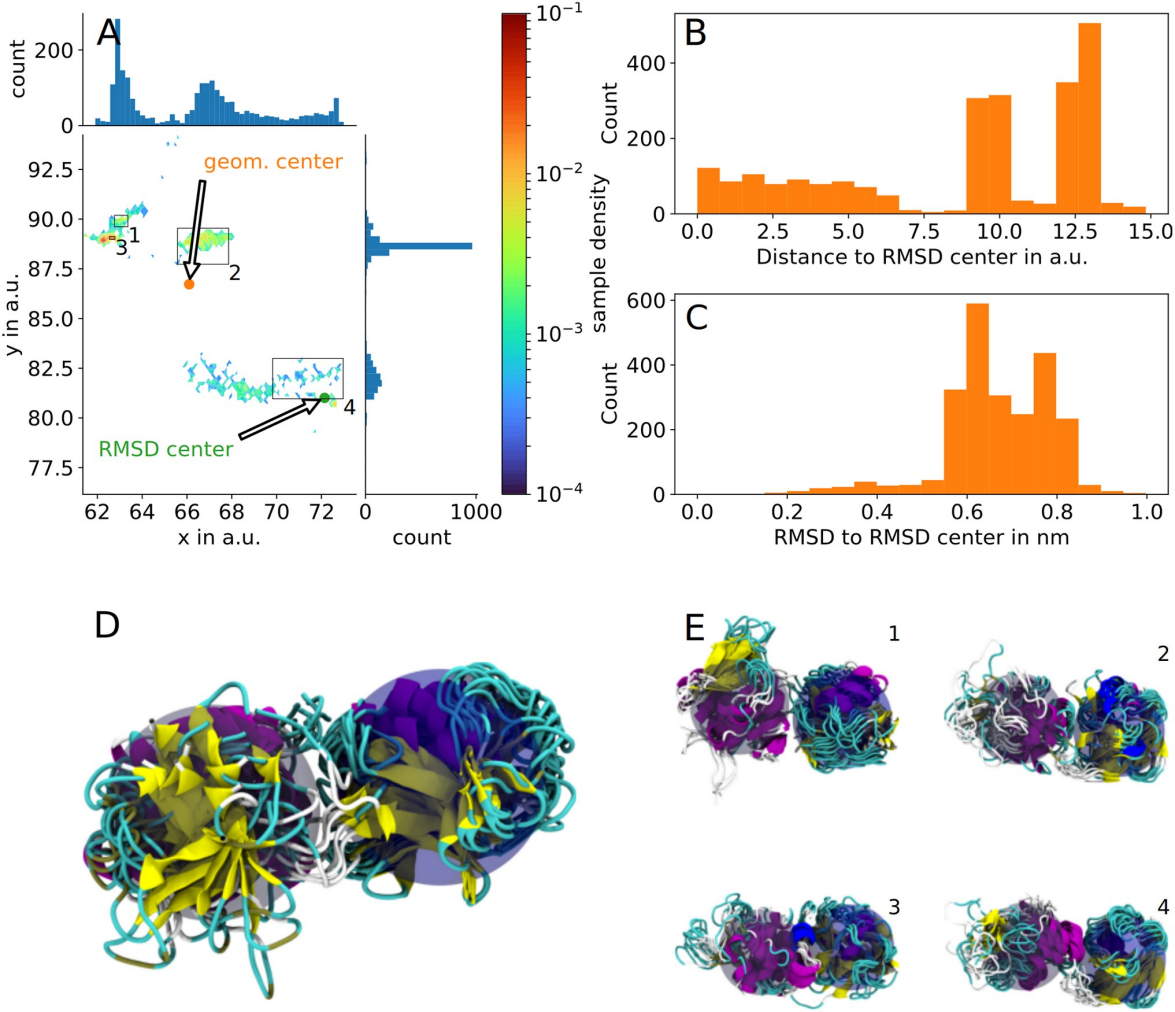
Refinement of non-uniform clusters via iterative HDBSCAN. An initial run of HDBSCAN combines all points in (A) into a single cluster. Rendering this cluster would result in a non-uniform bundle of structures (D). Furthermore, the spatial distribution in the low-dimensional space (B) and the distribution of RMSD values (C) indicate, that there are multiple sub-clusters that could be resolved by subsequent clustering. Using a lower minimal cluster size parameter the larger cluster is decomposed into smaller clusters (1–4) and the renderings of these regions (E 1–4) also show greater uniformity. Renders show the histone subunit (blue translucent sphere) on the right side and the ubiquitin subunit (grey translucent sphere) on the left side.

If for any given cluster at least one of the listed conditions was satisfied, this cluster was not assigned at that clustering iteration and points belonging to it were put back into the pool of all possible points to be resolved by subsequent (smaller cluster size) clustering (an example of such a cluster is shown in Fig 8).

The resulting 496 clusters were then used as representative sub-states of HUb to be evaluated further.

### Fitting HUb into the chromatosome

To get a measure of how well a given HUb structure “fits” into a parent chromatosome we developed an algorithm using the geometric properties of the HUb chromatosome system. Our interpenetration and scoring algorithm (ISA) uses the three-dimensional histogram of atomic positions of a nucleosome as its basis. The nucleosome coordinates are taken from one of the already published chromatosome structures (Table 1). The simulated HUb structures are now positioned into the nucleosome by superimposing the histone subunit on the coordinates of the reference linker histone using RMSD minimization. The Ub subunit will now possibly intersect with some parts of the nucleosome because the nucleosome was not present during the simulations. The extent of this clash (score) is now estimated by adding-up where the Ub subunit intersects with the nucleosome. This is done by binning the atomic coordinates of the nucleosome, creating a 3D histogram of atom counts. Every atom of the Ub subunit contributes to the score with the value of the bin it intersects and the score is calculated as the sum of the per-atom bin values.

An important factor to consider for this histogram (and histograms in general) is the number of bins. The larger the number of bins, the more “empty space” will be present in the 3D histogram. This creates a problem: The smaller the size of the bins, the more zero-count bins are in the 3D histogram. However, a fine binning would be beneficial to accurately model the surface of the nucleosome. To overcome this problem, we decided to implement a cumulative histogram in the scoring process. Here, eight separate cumulative histograms starting from the eight corners of the nucleosome’s bounding box are constructed. Following the diagonal of the bounding box each bin is assigned the number of the contained atoms plus the values assigned to its preceding and neighboring bins (see S6 Fig for examples in 1D and S7 Fig for 2D spaces). These 8 cumulative histograms are merged by assigning every bin the respective lowest value of the 8 cumulative histograms. The resulting scoring histogram captures the surface of the nucleosome via a fine binning, while still penalizing any Ub atoms that reach into the nucleosomal core with a high score. The scoring histogram is then min-max normalized onto the range [0, 1] to make values of scores more intuitive. At the end a single value is obtained for a given HUb (ligand) and nucleosome (receptor) pose.

With this new algorithm we were able to score all 483 983 conformations sampled by MD in record time (details are discussed in Results: Scoring HUb conformations into nucleosome structures), so no pre-screening or “cherry-picking” had to be conducted. Scores were calculated by superposing a given HUb conformation using the python package MDTraj’s superpose functionality and the Cα atoms of the crystal structure’s linker histones (Table 1). [66] If the number of Cα atoms of HUb (PDB 1GHC) did not match the number of the crystal structure’s linker histone a sequence alignment with T-COFFEE [68] was carried out beforehand. Given the huge size of both the molecular system and the dataset we designed the ISA to be particularly efficient by also parallelizing it using python’s joblib [69] package.

**Fig 9:**
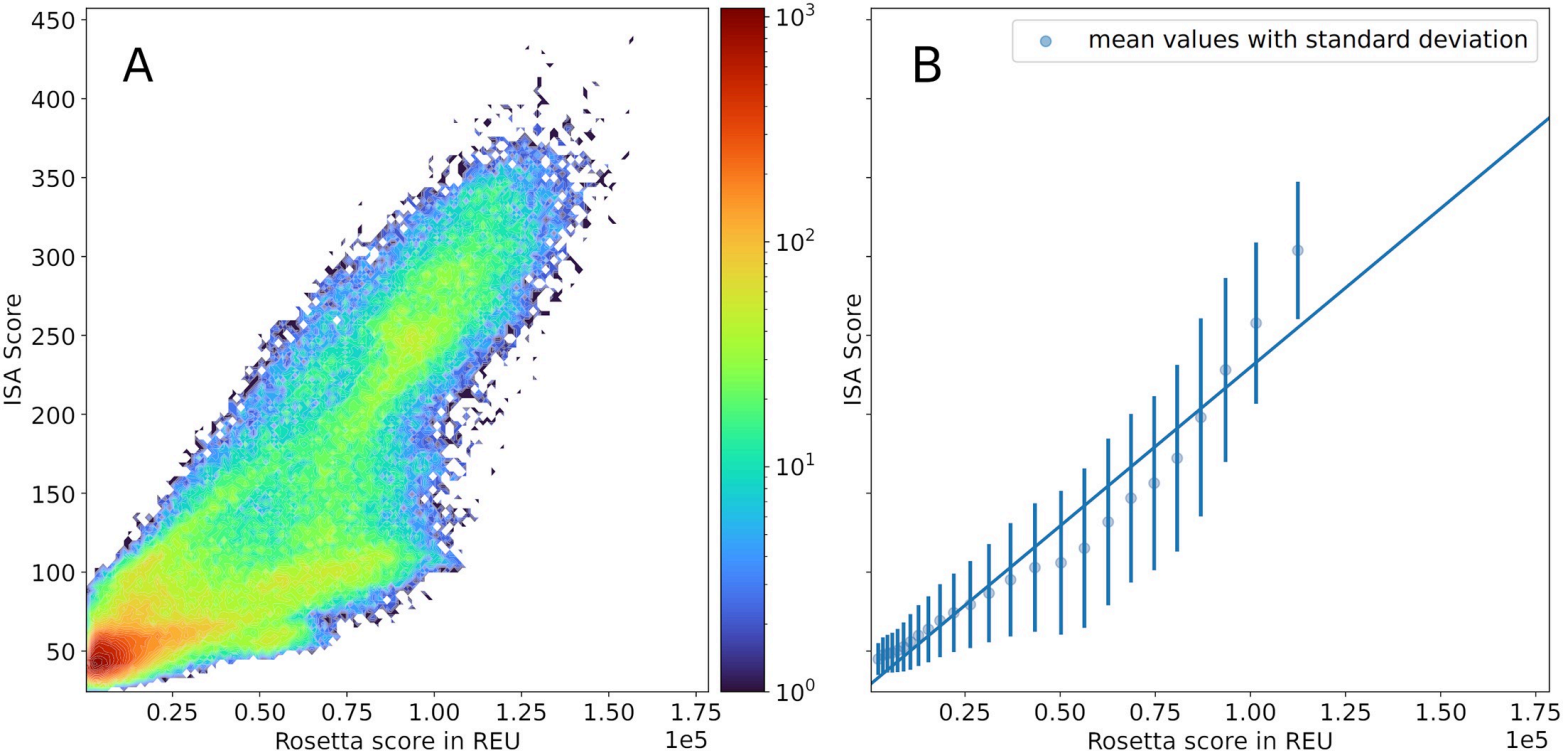
Comparison of scores obtained by our interpenetration and scoring algorithm (ISA) and ROSETTA for a subset of structures. We chose a random subset of structures from the expansion scheme simulations to score using ROSETTA and ISA. Rosetta scores range from −250 to ≈ 18 000 Rosetta Energy Units (REU). ROSETTA’s scoring functions with default weights support our claim that many structures exhibit lower scores, as the most structures can be found for lower scores where a high bin count can be observed (A). Our ISA algorithm correlates with the much more sophisticated ROSETTA algorithm with a Pearson correlation coefficient of 0.88 (B). However, our algorithm was 1000 times faster than pyROSETTA (both algorithms have been parallelized on a per-structure basis using the Python package joblib [69]).

Still, a fast algorithm needs to yield usable results, so we validate our novel and purely geometric approach to scoring with tried and tested scoring functions from ROSETTA [70, 71] (we used pyROSETTA, see S3 Text for more details). We were able to calculate ISA scores at a speed of 140 poses per second. In contrast, pyROSETTA took ≈ 10s per pose, which makes ISA roughly 1000 times faster than pyROSETTA. Although ISA does not use sophisticated potentials to model attractive and repulsive forces, a satisfying correlation of 0.88 to pyROSETTA (see Fig 9) was achieved.

#### Alignment of chromatosome structures into tetranucleosome

Chromatin contains many chromatosomes stacked in an orderly fashion and not just a single H1-nucleosome complex, so we also employed the ISA on a tetranucleosome array (PDB 1ZBB) published by Schalch et al. [4] This structure lacks the linker histones and thus we used a sequence and RMSD alignment of 1ZBB’s core histones with the core histones of 5NL0 [37] and the off-dyad chromatosome structure. For this we aligned the sequences of the 8 core histones with T-COFFEE and chose the longest uninterrupted residue sequence for RMSD alignment using MDTraj. Some additional adjustments had to be carried out to prevent the linker histone from clashing with the DNA of 1ZBB by manually adjusting the rotation of the chromatosomes around the third principal axis (S3 Table). Using these modelled linker histone positions we could directly use the ISA to get scores for HUb poses in the tetranucleosome array.

## Supporting information

S1 Text Supplementary Information 1. This file contains further details about the MD simulation setup: starting structures, initial simulations, expansion scheme implementation, MD parameters, long MD trajectories.
(PDF)

S2 Text Supplementary Information 2. This file contains further details about dimensionality reduction procedure with sketch-map: procedure and parameters, individual sketch-map projections and comparison of high-dimensional collective variables, high RMSD variance region.
(PDF)

S3 Text Supplementary Information 3. This file contains further details about ROSETTA scoring.
(PDF)

S1 Table Overview of the 50 largest clusters. The *hdbscan id* refers to the cluster id, assigned by the HDBSCAN algorithm. *cluster size* refers to the number of actual HUb conformations in this cluster. The percentages for the different linkage types give the composition of this cluster dependent on the HUb variants. *% of combined ensemble* relates the number of HUb conformations in this cluster with all simulated/sampled conformations. Following are values for ISA and ROSETTA scores. The internal RMSD was calculated by first finding the RMSD centroid of a cluster by finding the argmin of a pairwise RMSD distance matrix and then choosing that centroid as a reference for the RMSD calculations of internal RMSD. Given here are the mean (μ) and standard deviation (σ) in nm.
(PDF)

S2 Table Overview over the composition and scores of the ubiquitylation sites. All references to a score mean the ISA score. Clusters were selected by choosing clusters with the lowest mean ISA score and at least 90% composition of the respective ubiquitylation sit.
(PDF)

S3 Table Manual adjustments to create hypothetical linker histone positions in the tetranucleosome array 1ZBB. The reference chromatosomes pdb 5NL0 and the off-dyad structure were manually adjusted to allow their linker histone to fit into the tetranucleosome array of pdb 1ZBB. In the following table these adjustments are listed. “Core” refers to one of the cores of 1ZBB, where core1 has the lower segid of the first frame of the biological assembly and core4 has the higher segid of the second frame of the biological assembly.
(PDF)

S1 Fig Center of geometry distances of the initial long simulations and evolution of secondary structure motifs of one of these simulations. Time evolution of the center of geometry distances between the Ub-subunit and the H1-subunit of nine 1 μs simulations (A). Simulation names are composed of ubiquitylated lysine and starting χ_3_ angle (KXXX_χ_3_). The inset figure shows the same data in a 0–100 ns interval. Raw distances are transparent. Running averages over 7.5 ns are opaque. After relaxation (until ≈ 25 ns) the center of geometry stays similar during the evolution of the whole simulation. K47Ub with χ_3_ = 20° exhibits larger variations at the beginning, but approaches an equilibrium after that. The secondary structure motifs of this simulation also exhibit convergence of the structure (B).
(TIF)

S2 Fig Sketch-map projections of the initial simulations of K30Ub and K47Ub with RMSD evolution as inset figures. Exemplary sketch-map projections of initial simulations of K47Ub using SASA-CVs (A) and K30Ub using RMD-CVs (B). The color code indicates the starting χ_3_ angle of the respective ubiquitylated lysine residue. Similar angles have similar color saturation. Inset figures show RMSD evolution of selected trajectories. Larger RMSD deviations result in disjoint, scattered points. Smaller RMSD deviations yield cohesive patches. The simulation of K47Ub with a starting angle of χ_3_ = 0° exhibits little change in its RMSD after relaxation, which results in a densely populated patch (circle in (A)). Arrows in and annotated distances (B) connect the first and last point of the simulations with starting angle χ_3_ = 80° and χ_3_ = 120° and indicate the time evolution of these trajectories. The RMSD of the structures from K30Ub with an angle of χ_3_ = 120° exhibits a gradual increase, which is also traced in the sketch-map projection.
(TIF)

S3 Fig Individual sketch-map projections of the six variants during the expansion scheme colored by starting angle. K47Ub and K51Ub were projected using the SASA-CVs (Eq (1)) as input CVs for sketch-map. The other 4 variants were projected using the RMD-CVs (Eq (2)). Points are colored according to starting χ_3_ angle of the ubiquitylated lysine residue. A cyclic colormap was chosen to represent the periodic nature of dihedral angles.
(TIF)

S4 Fig Comparison of RMD-CVs and SASA-CVs for the projection of the whole dataset. For the top images (A-B) the RMD-CVs have been used as high-dimensional input for sketch-map. The lower images (C-D) were created by projecting the SASA-CVs. (A) and (C) are colored according to the ubiquitylation site of the variant. (B) and (D) are colored according to the mean RMSD distance to the subunit’s respective crystal structure (PDB ID 1GHC and 1UBQ). The RMD-CVs are not as suited as the SASA-CVs to project the conformational space of the six HUb proteins into the same low-dimensional map. More disorder for the top images can be observed than the for the bottom images, which contained the SASA-CVs in their creation. Thus the SASA-CVs were chosen to use as the high-dimensional collective variables for projecting the conformational space of all six variants into the same low-dimensional map.
(TIF)

S5 Fig Extracted structures from the high-density patch in the low-dimensional projection. In (A) the low-dimensional sketch-map projections (x and y coordinates) of all six variants using the SASA-CVs (Eq (1)) were plotted as a density map (colormap on the right). The high-density region is highlighted with a rectangle. In (B) 100 protein conformations originating from this region are shown. sketch-map was not able to separate these different conformations or even push them to the fringes of the projection map. For further analyses this region was excluded.
(TIF)

S6 Fig 1D example of cumulative histograms used in ISA. The 1D bounding box ranges from 0 to 5. Two cumulative histograms are created from a base histogram (blue). One ascending (orange), the other one descending (green). Both are visualized as smoothed curves. The final histogram which is then used for scoring is obtained by choosing the minimal value of either of the two cumulative histograms (red).
(TIF)

S7 Fig 2D example of ISA’s cumulative histograms with more points. In (A) 500 points have been placed in a ring with radius *r* = 3 ± *N*(*μ* = 0, *σ* = 0.2), where *N* is a standard normal distribution, around the origin. A normal histogram counting the number of points in a predefined set of bins is shown in (B), where yellow denotes highly occupied bins, purple empty bins. In (C) one of the four histograms obtained by walking from the lower left corner to the upper right is displayed. This results in some smoothing and “smearing” to the top right. In (D) the histogram for scoring is constructed by choosing the lowest value from the 4 cumulative histograms per bin. This histogram is used in ISA to determine the score of a given HUb-chromatosome pose.
(TIF)

S8 Fig Comparison of all ISA scores for monochromatosomes. The simulations of the six linkage types (K30 to K63) were placed into the four parent chromatosomes (5NL0, 5WCU, 4QLC and the off-dyad structure). In (A) the scores of all poses are visualized. The count reaches as high as 150 000 because all ubiquitylation variants and chromatosomes are combined. The four subfigures (B-E) give the per ubiquitylation variant score for the four chromatosomes 4QLC, 5NL0, 5WCU, and off-dyad respectively. It can be seen, that K56Ub (purple) tends to display higher scores after being placed into the off-dyad chromatosome (E) than for the 4QLC chromatosome (B). The remaining six subfigures (F-K) give the per chromatosome score for the six ubiquitylation variants K30Ub, K41Ub, K47Ub, K51Ub, K56Ub, and K63Ub, respectively. Here, it can be seen that the scores for the chromatosomes 5WCU and 4QLC (green and dark purple) are very similar. They almost coincide for all ubiquitylation variants. Furthermore, K56Ub (is very unfavorably positioned for the off-dyad chromatosome (yellow).
(TIF)

S9 Fig Best fitting clusters per ubiquitylation site using 5NL0 as the parent chromatosome. From all clusters found by iterative HDBSCAN clustering the cluster with the lowest ISA score containing at least 90% of a specific HUb variant is chosen. 20 equally spaced frames of these clusters are then placed into the parent 5NL0 chromatosome structure and visualized here. All of them have the Ub subunit pointing downward. For scores of other clusters refer to S1 Table.
(TIF)

## References

[1] Olins AL, Olins DE. Spheroid chromatin units (*ν* bodies). Science. 1974;183(4122):330–332. doi: 10.1126/science.183.4122.330 4128918

[2] Kornberg RD. Chromatin structure: a repeating unit of histones and DNA. Science. 1974;184(4139):868–871. doi: 10.1126/science.184.4139.868 4825889

[3] Carruthers L, Bednar J, Woodcock C, Hansen J. Linker histones stabilize the intrinsic salt-dependent folding of nucleosomal arrays: mechanistic ramifications for higher-order chromatin folding. Biochemistry. 1998;37(42):14776–14787. doi: 10.1021/bi981684e 9778352

[4] Schalch T, Duda S, Sargent DF, Richmond TJ. X-ray structure of a tetranucleosome and its implications for the chromatin fibre. Nature. 2005;436(7047):138–141. doi: 10.1038/nature03686 16001076

[5] Robinson PJ, Fairall L, Huynh VA, Rhodes D. EM measurements define the dimensions of the “30-nm” chromatin fiber: evidence for a compact, interdigitated structure. Proceedings of the National Academy of Sciences. 2006;103(17):6506–6511. doi: 10.1073/pnas.0601212103 16617109 PMC1436021

[6] Kruithof M, Chien FT, Routh A, Logie C, Rhodes D, Van Noort J. Single-molecule force spectroscopy reveals a highly compliant helical folding for the 30-nm chromatin fiber. Nature structural & molecular biology. 2009;16(5):534–540. doi: 10.1038/nsmb.1590 19377481

[7] Maeshima K, Imai R, Tamura S, Nozaki T. Chromatin as dynamic 10-nm fibers. Chromosoma. 2014;123(3):225–237. doi: 10.1007/s00412-014-0460-2 24737122 PMC4031381

[8] Norouzi D, Zhurkin VB. Topological polymorphism of the two-start chromatin fiber. Biophysical journal. 2015;108(10):2591–2600. doi: 10.1016/j.bpj.2015.04.015 25992737 PMC4457244

[9] Luger K, Dechassa ML, Tremethick DJ. New insights into nucleosome and chromatin structure: an ordered state or a disordered affair? Nature reviews Molecular cell biology. 2012;13(7):436–447. doi: 10.1038/nrm3382 22722606 PMC3408961

[10] Zhou BR, Feng H, Kato H, Dai L, Yang Y, Zhou Y, et al. Structural insights into the histone H1-nucleosome complex. Proceedings of the National Academy of Sciences. 2013;110(48):19390–19395. doi: 10.1073/pnas.1314905110 24218562 PMC3845106

[11] Bednar J, Hamiche A, Dimitrov S. H1–nucleosome interactions and their functional implications. Biochimica et Biophysica Acta (BBA)—Gene Regulatory Mechanisms. 2016;1859(3):436–443. doi: 10.1016/j.bbagrm.2015.10.012 26477489

[12] Sun ZW, Allis CD. Ubiquitination of histone H2B regulates H3 methylation and gene silencing in yeast. Nature. 2002;418(6893):104–108. doi: 10.1038/nature00883 12077605

[13] Zentner GE, Henikoff S. Regulation of nucleosome dynamics by histone modifications. Nature structural & molecular biology. 2013;20(3):259. doi: 10.1038/nsmb.2470 23463310

[14] Kim W, Bennett EJ, Huttlin EL, Guo A, Li J, Possemato A, et al. Systematic and quantitative assessment of the ubiquitin-modified proteome. Molecular cell. 2011;44(2):325–340. doi: 10.1016/j.molcel.2011.08.025 21906983 PMC3200427

[15] Wagner SA, Beli P, Weinert BT, Nielsen ML, Cox J, Mann M, et al. A proteome-wide, quantitative survey of in vivo ubiquitylation sites reveals widespread regulatory roles. Molecular & Cellular Proteomics. 2011;10(10). doi: 10.1074/mcp.M111.013284 21890473 PMC3205876

[16] Danielsen JM, Sylvestersen KB, Bekker-Jensen S, Szklarczyk D, Poulsen JW, Horn H, et al. Mass spectrometric analysis of lysine ubiquitylation reveals promiscuity at site level. Molecular & Cellular Proteomics. 2011;10(3). doi: 10.1074/mcp.M110.003590 21139048 PMC3047152

[17] Chang L, Shen L, Zhou H, Gao J, Pan H, Zheng L, et al. ITCH nuclear translocation and H1. 2 polyubiquitination negatively regulate the DNA damage response. Nucleic acids research. 2019;47(2):824–842. doi: 10.1093/nar/gky1199 30517763 PMC6344871

[18] Thorslund T, Ripplinger A, Hoffmann S, Wild T, Uckelmann M, Villumsen B, et al. Histone H1 couples initiation and amplification of ubiquitin signalling after DNA damage. Nature. 2015;527(7578):389–393. doi: 10.1038/nature15401 26503038

[19] Ding X, Lin X, Zhang B. Stability and folding pathways of tetra-nucleosome from six-dimensional free energy surface. Nature communications. 2021;12(1):1–9. doi: 10.1038/s41467-021-21377-z 33597548 PMC7889939

[20] Sharma S, Ding F, Dokholyan NV. Multiscale modeling of nucleosome dynamics. Biophysical journal. 2007;92(5):1457–1470. doi: 10.1529/biophysj.106.094805 17142268 PMC1796817

[21] Arya G, Schlick T. Role of histone tails in chromatin folding revealed by a mesoscopic oligonucleosome model. Proceedings of the National Academy of Sciences. 2006;103(44):16236–16241. doi: 10.1073/pnas.0604817103 17060627 PMC1637566

[22] Brandani GB, Tan C, Takada S. The kinetic landscape of nucleosome assembly: a coarse-grained molecular dynamics study. PLoS computational biology. 2021;17(7):e1009253. doi: 10.1371/journal.pcbi.1009253 34314440 PMC8345847

[23] Huertas J, Schöler HR, Cojocaru V. Histone tails cooperate to control the breathing of genomic nucleosomes. PLoS computational biology. 2021;17(6):e1009013. doi: 10.1371/journal.pcbi.1009013 34081696 PMC8174689

[24] Allan J, Hartman P, Crane-Robinson C, Aviles F. The structure of histone H1 and its location in chromatin. Nature. 1980;288(5792):675–679. doi: 10.1038/288675a0 7453800

[25] Pruss D, Bartholomew B, Persinger J, Hayes J, Arents G, Moudrianakis EN, et al. An asymmetric model for the nucleosome: a binding site for linker histones inside the DNA gyres. Science. 1996;274(5287):614–617. doi: 10.1126/science.274.5287.614 8849453

[26] Zhou YB, Gerchman SE, Ramakrishnan V, Travers A, Muyldermans S. Position and orientation of the globular domain of linker histone H5 on the nucleosome. Nature. 1998;395(6700):402–405. doi: 10.1038/26521 9759733

[27] Syed SH, Goutte-Gattat D, Becker N, Meyer S, Shukla MS, Hayes JJ, et al. Single-base resolution mapping of H1–nucleosome interactions and 3D organization of the nucleosome. Proceedings of the National Academy of Sciences. 2010;107(21):9620–9625. doi: 10.1073/pnas.1000309107 20457934 PMC2906896

[28] Woods DC, Wereszczynski J. Elucidating the influence of linker histone variants on chromatosome dynamics and energetics. Nucleic acids research. 2020;48(7):3591–3604. doi: 10.1093/nar/gkaa121 32128577 PMC7144933

[29] Öztürk MA, De M, Cojocaru V, Wade RC. Chromatosome structure and dynamics from molecular simulations. Annual review of physical chemistry. 2020;71:101–119. doi: 10.1146/annurev-physchem-071119-040043 32017651

[30] Sridhar A, Farr SE, Portella G, Schlick T, Orozco M, Collepardo-Guevara R. Emergence of chromatin hierarchical loops from protein disorder and nucleosome asymmetry. Proceedings of the National Academy of Sciences. 2020;117(13):7216–7224. doi: 10.1073/pnas.1910044117 32165536 PMC7132128

[31] Davey CA, Sargent DF, Luger K, Maeder AW, Richmond TJ. Solvent mediated interactions in the structure of the nucleosome core particle at 1.9 Å resolution. Journal of molecular biology. 2002;319(5):1097–1113. doi: 10.1016/S0022-2836(02)00386-8 12079350

[32] Rychkov GN, Ilatovskiy AV, Nazarov IB, Shvetsov AV, Lebedev DV, Konev AY, et al. Partially assembled nucleosome structures at atomic detail. Biophysical journal. 2017;112(3):460–472. doi: 10.1016/j.bpj.2016.10.041 28038734 PMC5300784

[33] Izadi S, Anandakrishnan R, Onufriev AV. Implicit solvent model for million-atom atomistic simulations: insights into the organization of 30-nm chromatin fiber. Journal of chemical theory and computation. 2016;12(12):5946–5959. doi: 10.1021/acs.jctc.6b00712 27748599 PMC5649046

[34] Fan Y, Korolev N, Lyubartsev AP, Nordenskiöld L. An advanced coarse-grained nucleosome core particle model for computer simulations of nucleosome-nucleosome interactions under varying ionic conditions. PLoS One. 2013;8(2):e54228. doi: 10.1371/journal.pone.0054228 23418426 PMC3572162

[35] Bendandi A, Dante S, Zia SR, Diaspro A, Rocchia W. Chromatin compaction multiscale modeling: a complex synergy between theory, simulation, and experiment. Frontiers in Molecular Biosciences. 2020;7:15. doi: 10.3389/fmolb.2020.00015 32158765 PMC7051991

[36] Wu H, Dalal Y, Papoian GA. Binding dynamics of disordered linker histone H1 with a nucleosomal particle. Journal of molecular biology. 2021;433(6):166881. doi: 10.1016/j.jmb.2021.166881 33617899 PMC9272445

[37] Bednar J, Garcia-Saez I, Boopathi R, Cutter AR, Papai G, Reymer A, et al. Structure and dynamics of a 197 bp nucleosome in complex with linker histone H1. Molecular cell. 2017;66(3):384–397. doi: 10.1016/j.molcel.2017.04.012 28475873 PMC5508712

[38] Cerf C, Lippens G, Muyldermans S, Segers A, Ramakrishnan V, Wodak SJ, et al. Homo-and heteronuclear two-dimensional NMR studies of the globular domain of histone H1: sequential assignment and secondary structure. Biochemistry. 1993;32(42):11345–11351. doi: 10.1021/bi00093a011 8218199

[39] Höllmüller E, Geigges S, Niedermeier ML, Kammer KM, Kienle SM, Rösner D, et al. Site-specific ubiquitylation acts as a regulator of linker histone H1. Nature Communications. 2021;12(1):1–15. doi: 10.1038/s41467-021-23636-5 34108453 PMC8190259

[40] Fierz B, Chatterjee C, McGinty RK, Bar-Dagan M, Raleigh DP, Muir TW. Histone H2B ubiquitylation disrupts local and higher-order chromatin compaction. Nature chemical biology. 2011;7(2):113–119. doi: 10.1038/nchembio.501 21196936 PMC3078768

[41] Höllmüller E, Greiner K, Kienle SM, Scheffner M, Marx A, Stengel F. Interactome of Site-Specifically Acetylated Linker Histone H1. Journal of Proteome Research. 2021;20(9):4443–4451. doi: 10.1021/acs.jproteome.1c00396 34351766

[42] Zhou BR, Jiang J, Feng H, Ghirlando R, Xiao TS, Bai Y. Structural mechanisms of nucleosome recognition by linker histones. Molecular cell. 2015;59(4):628–638. doi: 10.1016/j.molcel.2015.06.025 26212454 PMC4546531

[43] Zhou BR, Jiang J, Ghirlando R, Norouzi D, Yadav KS, Feng H, et al. Revisit of reconstituted 30-nm nucleosome arrays reveals an ensemble of dynamic structures. Journal of molecular biology. 2018;430(18):3093–3110. doi: 10.1016/j.jmb.2018.06.020 29959925 PMC6204201

[44] Wang S, Vogirala VK, Soman A, Berezhnoy NV, Liu ZB, Wong AS, et al. Linker histone defines structure and self-association behaviour of the 177 bp human chromatosome. Scientific Reports. 2021;11(1):1–16. doi: 10.1038/s41598-020-79654-8 33432055 PMC7801413

[45] Arimura Y, Shih RM, Froom R, Funabiki H. Structural features of nucleosomes in interphase and metaphase chromosomes. Molecular Cell. 2021;81(21):4377–4397. doi: 10.1016/j.molcel.2021.08.010 34478647 PMC8571072

[46] Zhou BR, Feng H, Kale S, Fox T, Khant H, de Val N, et al. Distinct structures and dynamics of chromatosomes with different human linker histone isoforms. Molecular Cell. 2021;81(1):166–182. doi: 10.1016/j.molcel.2020.10.038 33238161 PMC7796963

[47] Thåström A, Bingham L, Widom J. Nucleosomal locations of dominant DNA sequence motifs for histone–DNA interactions and nucleosome positioning. Journal of molecular biology. 2004;338(4):695–709. doi: 10.1016/j.jmb.2004.03.032 15099738

[48] Correll SJ, Schubert MH, Grigoryev SA. Short nucleosome repeats impose rotational modulations on chromatin fibre folding. The EMBO journal. 2012;31(10):2416–2426. doi: 10.1038/emboj.2012.80 22473209 PMC3364735

[49] Rösner D. Chemical mono-ubiquitylation of linker histone H1.2 by combining unnatural amino acids with click chemistry: Synthesis and structural studies [Ph.D. thesis]. University of Konstanz. Universitätsstr. 10, D-78464 Konstanz, Germany; 2015. Available from: http://nbn-resolving.de/urn:nbn:de:bsz:352-0-326144.

[50] Ramakrishnan V, Finch J, Graziano V, Lee P, Sweet R. Crystal structure of globular domain of histone H5 and its implications for nucleosome binding. Nature. 1993;362(6417):219–223. doi: 10.1038/362219a0 8384699

[51] Kukharenko O, Sawade K, Steuer J, Peter C. Using dimensionality reduction to systematically expand conformational sampling of intrinsically disordered peptides. Journal of Chemical Theory and Computation. 2016;12(10):4726–4734. doi: 10.1021/acs.jctc.6b00503 27588692

[52] Ceriotti M, Tribello GA, Parrinello M. Simplifying the representation of complex free-energy landscapes using sketch-map. Proceedings of the National Academy of Sciences. 2011;108(32):13023–13028. doi: 10.1073/pnas.1108486108 21730167 PMC3156203

[53] Berg A, Peter C. Simulating and analysing configurational landscapes of protein–protein contact formation. Interface focus. 2019;9(3):20180062. doi: 10.1098/rsfs.2018.0062 31065336 PMC6501351

[54] McInnes L, Healy J, Astels S. hdbscan: Hierarchical density based clustering. The Journal of Open Source Software. 2017;2(11). doi: 10.21105/joss.00205

[55] Arya G, Zhang Q, Schlick T. Flexible histone tails in a new mesoscopic oligonucleosome model. Biophysical journal. 2006;91(1):133–150. doi: 10.1529/biophysj.106.083006 16603492 PMC1479056

[56] Humphrey W, Dalke A, Schulten K. VMD: visual molecular dynamics. Journal of molecular graphics. 1996;14(1):33–38. doi: 10.1016/0263-7855(96)00018-5 8744570

[57] Ghoneim M, Fuchs HA, Musselman CA. Histone tail conformations: a fuzzy affair with DNA. Trends in biochemical sciences. 2021;46(7):564–578. doi: 10.1016/j.tibs.2020.12.012 33551235 PMC8195839

[58] Andrews FH, Strahl BD, Kutateladze TG. Insights into newly discovered marks and readers of epigenetic information. Nature chemical biology. 2016;12(9):662–668. doi: 10.1038/nchembio.2149 27538025 PMC5116920

[59] Li Z, Kono H. Distinct roles of histone H3 and H2A tails in nucleosome stability. Scientific reports. 2016;6(1):31437. doi: 10.1038/srep31437 27527579 PMC4985630

[60] Stützer A, Liokatis S, Kiesel A, Schwarzer D, Sprangers R, Söding J, et al. Modulations of DNA contacts by linker histones and post-translational modifications determine the mobility and modifiability of nucleosomal H3 tails. Molecular cell. 2016;61(2):247–259. doi: 10.1016/j.molcel.2015.12.015 26778125

[61] Vijay-Kumar S, Bugg CE, Cook WJ. Structure of ubiquitin refined at 1.8 Åresolution. Journal of molecular biology. 1987;194(3):531–544. doi: 10.1016/0022-2836(87)90679-6 3041007

[62] Pettersen EF, Goddard TD, Huang CC, Couch GS, Greenblatt DM, Meng EC, et al. UCSF Chimera—a visualization system for exploratory research and analysis. Journal of computational chemistry. 2004;25(13):1605–1612. doi: 10.1002/jcc.20084 15264254

[63] Van Der Spoel D, Lindahl E, Hess B, Groenhof G, Mark AE, Berendsen HJ. GROMACS: fast, flexible, and free. Journal of computational chemistry. 2005;26(16):1701–1718. doi: 10.1002/jcc.20291 16211538

[64] Schmid N, Eichenberger A, Choutko A, Riniker S, Winger M, Mark A, et al. testing of the GROMOS force-field versions: 54A7 and 54B7 Eur. Biophys J. 2011;40:843–856. 10.1007/s00249-011-0700-9 21533652

[65] Berg A, Kukharenko O, Scheffner M, Peter C. Towards a molecular basis of ubiquitin signaling: A dual-scale simulation study of ubiquitin dimers. PLOS Computational Biology. 2018;14(11):1–14. doi: 10.1371/journal.pcbi.1006589 30444864 PMC6268000

[66] McGibbon RT, Beauchamp KA, Harrigan MP, Klein C, Swails JM, Hernández CX, et al. MDTraj: A Modern Open Library for the Analysis of Molecular Dynamics Trajectories. Biophysical Journal. 2015;109(8):1528–1532. doi: 10.1016/j.bpj.2015.08.015 26488642 PMC4623899

[67] Maurus S, Plant C. Skinny-dip: clustering in a sea of noise. In: Proceedings of the 22nd ACM SIGKDD international conference on Knowledge discovery and data mining; 2016. p. 1055–1064.

[68] Notredame C, Higgins DG, Heringa J. T-Coffee: A novel method for fast and accurate multiple sequence alignment. Journal of molecular biology. 2000;302(1):205–217. doi: 10.1006/jmbi.2000.4042 10964570

[69] Joblib Development Team. Joblib: running Python functions as pipeline jobs; 2020. Available from: https://joblib.readthedocs.io/.

[70] Leaver-Fay A, Tyka M, Lewis SM, Lange OF, Thompson J, Jacak R, et al. ROSETTA3: an object-oriented software suite for the simulation and design of macromolecules. In: Methods in enzymology. vol. 487. Elsevier; 2011. p. 545–574. 21187238 10.1016/B978-0-12-381270-4.00019-6 PMC4083816

[71] Kaufmann KW, Lemmon GH, DeLuca SL, Sheehan JH, Meiler J. Practically useful: what the Rosetta protein modeling suite can do for you. Biochemistry. 2010;49(14):2987–2998. doi: 10.1021/bi902153g 20235548 PMC2850155

