# Supplementary material for: Combining molecular dynamics simulations and scoring method to computationally model ubiquitylated linker histones in chromatosomes
Source: PLoS Comput Biol. 2023 Aug 1;19(8):e1010531. doi: 10.1371/journal.pcbi.1010531 (PMC10442151; doi:10.1371/journal.pcbi.1010531)
Supplement: S1 Text — This file contains further details about the MD simulation setup: starting structures, initial simulations, expansion scheme implementation, MD parameters, long MD trajectories. (PDF) [file pcbi.1010531.s001.pdf]

Supplementary Information 1 for:
Combining molecular dynamics simulations and scoring method to computationally model ubiquitylated linker histones in chromatosomes

S1 MD simulation set up

S1.1 Starting structures

S1.1.1 Initial long simulations

Starting structures for all simulations were generated by joining ubiquitin's C-terminus (G76 of PDB ID 1UBQ [1]) with K30, K41, K47, K51, K56, and K60 of avian linker histone H1 (PDB ID 1GHC [2]) using the program UCSF Chimera. [3] The connecting lysine and glycine were renamed to LYQ and GLQ, respectively. Two of lysine's hydrogens and one oxygen atom of glycine were deleted. For the long $\approx 1\,\mu s$ simulations the χ_3-angle of the ubiquitylated lysine was adjusted to the values given in Table A.

Table A. Long $\approx 1\,\mu s$ simulations carried out to study the system. After these simulations it became apparent, that the expansion scheme would be applicable here to generate more conformations in a shorter simulation time.

Ubiquitylation Site	χ_3 rotation in degree	Time in ps
K30	No long simulations	No long simulations
K41	No long simulations	No long simulations
K47	-100	1000000
K47	140	1000000
K47	20	1000000
K51	-120	1000000
K51	0	1000000
K51	120	1000000
K56	-120	1000000
K56	0	1000000
K56	120	1000000
K63	-100	1000000
K63	140	1000000
K63	20	1076839
K63	60	1076839

S1.1.2 Starting structures for expansion scheme

The χ_3-angle was adjusted in 20° steps to create 18 starting structures for each of the ubiquitylation sites K30, K41, K47, K51, K56, and K63. Some dihedral angles resulted in starting structures that could not be energy-minimized due to clashes of the two subunits. These starting angles were omitted, which led to some specific ubiquitylation-site angle combinations not being present in the simulation set. These

initial simulations then were fed into the expansion scheme. For every variant 20 new simulations from points selected in sketch-map space were started. These expansion simulations were started from the solvated system and underwent a short (0.2 ns) position-restrained simulation before conducting MD simulations, using the same parameters as in Section S1.2.

S1.2 MD parameters

The starting structures were fed into all-atom molecular dynamics simulations using the GROMACS program package. [4] The GROMOS54a7 [5] forcefield was modified to include the isopeptide connection (see Section S1.2.1). The starting structures for K30, K47, and K63 were put into a dodecahederal box with 0.8 nm distance to the walls, reducing the number of explicit solvent molecules needing to be simulated. The starting structures for K41, K51, and K63 were centered in a dodecahedral box with a volume of 700 nm^2. Periodic boundary conditions were implemented, proteins were solvated in SPC water [6], and charges were neutralized with Na^+ and Cl^- ions. The systems underwent standard steepest descent energy minimization and were equilibrated with the positions of the heavy atoms restrained for 0.2 ps. MD simulations employed the leapfrog integrator [7] with an integration step of 2 fs. The temperature was kept at 300 K using the velocity rescale algorithm [8] with a time constant of $\tau_t = 1.0$ ps. Pressure was kept at 101 325 Pa with the Berendsen barostat [9] with a time constant of $\tau_p = 1.0$ ps. Bond constraints were handled by the LINCS algorithm. [10] Long range electrostatics were calculated by the Particle Mesh Ewald Method [11] with a Fourier spacing of 0.12 nm and cubic interpolation. Coulomb, Van-der-Waals and the long neighbor list cutoffs were set to be 1.4 nm. The short neighbor list cutoff was set to 1 nm. Neighbor lists were updated every 10 steps. The simulations were carried out on the HPC resources bwUniCluster and bwForCluster MLS&WISO.Completed simulations were stripped of solvent and frames were extracted from the trajectory every 50 ps resulting in 401 frames per trajectory.

S1.2.1 Adding isopeptide bonds to the GROMOS54a7 forcefield

The GROMOS54a7 forcefield was changed following a procedure described by Berg et al. [12] The renamed lysine (LYQ) and glycine (GLQ) were added to the forcefield's `residuetypes.dat` and to `aminoacids.hdb`. Additionally, the sidechain nitrogen was renamed to NQ, because it needed different interaction potentials, than lysine "standard" nitrogen NZ. The isopeptide bond between LYQ-NQ and GLQ-C was defined in the forcefield's `specbond.dat`. The two new aminoacids were added to `aminoacids.rtp` using their non-iospeptide counterparts as base and removing unwanted atoms bonds, angles, dihedrals, and impropers. The termini of COQ were adjusted in `aminoacids.c.tdb`. The proteins were built with UCSF Chimera [3] and saved as pdb files. GROMACS' `pdb2gmx` program was used with the modified forcefield to create GROMACS topologies.

S1.3 Slow conformational exploration of initial long simulations

The ≈ 1 µs simulations described in Table A did not result in a sufficiently high sampling rate of the conformational space available to the variants (Fig S1). For this reason we decided to start more simulations and apply the expansion scheme to this system. The simulations obtained by this method exhibit not only greater variance in the distance between the center of geometry of the two subunits, but also greater distances which indicates that rare events occur more frequently.

References

1. Vijay-Kumar S, Bugg CE, Cook WJ. Structure of ubiquitin refined at 1.8 Åresolution. Journal of molecular biology. 1987;194(3):531–544.

2. Cerf C, Lippens G, Muyldermans S, Segers A, Ramakrishnan V, Wodak SJ, et al. Homo-and heteronuclear two-dimensional NMR studies of the globular domain of histone H1: sequential assignment and secondary structure. Biochemistry. 1993;32(42):11345–11351.

3. Pettersen EF, Goddard TD, Huang CC, Couch GS, Greenblatt DM, Meng EC, et al. UCSF Chimera—a visualization system for exploratory research and analysis. Journal of computational chemistry. 2004;25(13):1605–1612.

4. Van Der Spoel D, Lindahl E, Hess B, Groenhof G, Mark AE, Berendsen HJ. GROMACS: fast, flexible, and free. Journal of computational chemistry. 2005;26(16):1701–1718.

5. Schmid N, Eichenberger A, Choutko A, Riniker S, Winger M, Mark A, et al. testing of the GROMOS force-field versions: 54A7 and 54B7 Eur. Biophys J. 2011;40:843–856.

6. Berendsen HJ, Postma JP, van Gunsteren WF, Hermans J. Interaction models for water in relation to protein hydration. In: Intermolecular forces. Springer; 1981. p. 331–342.

7. Fincham D. Leapfrog rotational algorithms. Molecular Simulation. 1992;8(3-5):165–178.

8. Bussi G, Donadio D, Parrinello M. Canonical sampling through velocity rescaling. The Journal of chemical physics. 2007;126(1):014101.

9. Berendsen HJ, Postma Jv, van Gunsteren WF, DiNola A, Haak JR. Molecular dynamics with coupling to an external bath. The Journal of chemical physics. 1984;81(8):3684–3690.

10. Hess B, Bekker H, Berendsen HJ, Fraaije JG. LINCS: a linear constraint solver for molecular simulations. Journal of computational chemistry. 1997;18(12):1463–1472.

11. Darden T, York D, Pedersen L. Particle mesh Ewald: An N· log (N) method for Ewald sums in large systems. The Journal of chemical physics. 1993;98(12):10089–10092.

12. Berg A, Kukharenko O, Scheffner M, Peter C. Towards a molecular basis of ubiquitin signaling: A dual-scale simulation study of ubiquitin dimers. PLOS Computational Biology. 2018;14(11):1–14. doi:10.1371/journal.pcbi.1006589.
