# Supplementary material for: Combining molecular dynamics simulations and scoring method to computationally model ubiquitylated linker histones in chromatosomes
Source: PLoS Comput Biol. 2023 Aug 1;19(8):e1010531. doi: 10.1371/journal.pcbi.1010531 (PMC10442151; doi:10.1371/journal.pcbi.1010531)
Supplement: S2 Text — This file contains further details about dimensionality reduction procedure with sketch-map: procedure and parameters, individual sketch-map projections and comparison of high-dimensional collective variables, high RMSD variance region. (PDF) [file pcbi.1010531.s002.pdf]

Supplementary Information 2 for: Combining molecular dynamics simulations and scoring method to computationally model ubiquitylated linker histones in chromatosomes

S2 Dimensionality reduction with sketch-map

S2.1 General procedure

We explored two different CVs as input for dimensionality reduction with sketch-map [1]. K41HUb, K47Ub, K51Ub, and K63HUb were plugged into the expansion scheme using the SASA-CVs, while from K30Ub and K56Ub the RMD-CVs were used as high-dimensional input CVs for sketch-map. The parameters of the high- and low-dimensional sigmoid functions used to transform the high- and low-dimensional CV-space and sketch-map space are given in Table A

Table A. Sketch-map parameters used to project the SASA-CVs and the RMD-CVs.

High-D data	D	d	σ	A	B	a	b
SASA-CVs	304	2	6.0	10	3	2	3
RMD-CVs	75	2	1.5	5	2	1	2

For projecting the simulations of each variant separately, 1000 landmarks were selected using a farthest point sampling (`dimlandmark`). For the combined projection of all variants, 2000 landmarks were selected from the complete SASA-CV feature space. These landmarks were projected into 2D using sketch-map's simulated annealing script. One should note, that this iterative optimization procedure, is not always able to produce an optimal (or satisfactory) output for such high-dimensional and diverse data set. We have encountered two types of flaws. First, some points (from a total of 483 983 points 1306 were placed beyond the 150 a.u. of the main projection area, 357 were placed beyond 1000 a.u. and 5 were placed beyond 1 000 000 a.u.) were placed outside of the main projection area. For each of these points we selected the closest neighbor in the main projection area and placed near it at a random position within ±0.5 length units. Second, we also encountered a considerable number of points with identical (x, y) coordinates, which formed quite sharp density peaks. The structures inside those areas were very similar, but for the consistency of the analysis (without removing them density-based clustering is not able to find clusters), we removed those points from the data set for clustering and then reassigned the resulting cluster memberships.

S2.2 Comparison of high-dimensional collective variables

From the accumulated simulations a combined sketch-map projection was created using the SASA-CVs as high-dimensional input. The reason we decided against the

RMD-CVs is visualized in Fig S5. Using the RMD CVs, sketch-map was not able to separate the structures in the low-dimensional projection. This is especially drastic when comparing the RMSD distance to the crystal structure (Fig S5B and S5C).

S2.3 High RMSD variance region

Due to the amount of different structures and high-dimensionality of the initial space the sketch-map optimization was not always able to move the unique structures to the fringe regions of the landscape like it did when every variant is projected into its own low-dimensional landscape (Fig S4). In our most promising projection these unique structure can be found aggregated in a region of high RMSD variance (Section S2.3). Due to optimisation problems the sketch-map was placing some points at the exact same xy-coordinates. With this dataset, no density-based clustering could be conducted, as these xy-coordinate-duplicates would have been regarded as the density maximum and all other points would have been assigned as noise. Example of such high-density patch is shown in Fig S6. Thus, the data set made it necessary to first exclude the region of high density and high RMSD variance, then the xy-coordinate duplicates, run the density-based clustering before reassigning the xy-coordinate duplicates and assigning the high density, high RMSD variance region to the noise.

References

1. Ceriotti M, Tribello GA, Parrinello M. Simplifying the representation of complex free-energy landscapes using sketch-map. Proceedings of the National Academy of Sciences. 2011;108(32):13023–13028.
