# Supplementary material for: Combining molecular dynamics simulations and scoring method to computationally model ubiquitylated linker histones in chromatosomes
Source: PLoS Comput Biol. 2023 Aug 1;19(8):e1010531. doi: 10.1371/journal.pcbi.1010531 (PMC10442151; doi:10.1371/journal.pcbi.1010531)
Supplement: S3 Text — This file contains further details about ROSETTA scoring. (PDF) [file pcbi.1010531.s003.pdf]

Supplementary Information 3 for:
Combining molecular dynamics simulations and scoring method to computationally model ubiquitylated linker histones in chromatosomes

S3 ROSETTA scoring

For ROSETTA two input structures needed to be created. The receptor.pdb was created by removing the linker histone from the chromatosomes listed in the results section of the main text. The ligand.pdb was cleaned from the isopeptide LYQ and GLQ residues, because they would have caused errors in ROSETTA, and superposed using MDTraj. A scoring protocol (no dynamics, no refinement) was adapted from Evan H. Baugh's D020_Pose_scoring tutorial. [1] The weights of ROSETTA's scoring functions were left at their standard values. The comparison of ISA and ROSETTA was done on the 5NL0 chromatosome.

References

1. Leaver-Fay A, Tyka M, Lewis SM, Lange OF, Thompson J, Jacak R, et al. ROSETTA3: an object-oriented software suite for the simulation and design of macromolecules. In: Methods in enzymology. vol. 487. Elsevier; 2011. p. 545–574.
