# Supplementary material for: Combining molecular dynamics simulations and scoring method to computationally model ubiquitylated linker histones in chromatosomes
Source: PLoS Comput Biol. 2023 Aug 1;19(8):e1010531. doi: 10.1371/journal.pcbi.1010531 (PMC10442151; doi:10.1371/journal.pcbi.1010531)
Supplement: S1 Table — The hdbscan id refers to the cluster id, assigned by the HDBSCAN algorithm. cluster size refers to the number of actual HUb conformations in this cluster. The percentages for the different linkage types give the composition of this cluster dependent on the HUb variants. % of combined ensemble relates the number of HUb conformations in this cluster with all simulated/sampled conformations. Following are values for ISA and ROSETTA scores. The internal RMSD was calculated by first finding the RMSD centroid of a cluster by finding the argmin of a pairwise RMSD distance matrix and then choosing that centroid as a reference for the RMSD calculations of internal RMSD. Given here are the mean (μ) and standard deviation (σ) in nm. (PDF) [file pcbi.1010531.s004.pdf]

Table S1: Overview of the 50 largest clusters. The *hdbscan id* refers to the cluster id, assigned by the HDBSCAN algorithm. *cluster size* refers to the number of actual HUb conformations in this cluster. The percentages for the different linkage types give the composition of this cluster dependent on the HUb variants. *% of combined ensemble* relates the number of HUb conformations in this cluster with all simulated/sampled conformations. Following are values for ISA and ROSETTA scores. The internal RMSD was calculated by first finding the RMSD centroid of a cluster by finding the argmin of a pairwise RMSD distance matrix and then choosing that centroid as a reference for the RMSD calculations of internal RMSD. Given here are the mean (μ) and standard deviation (σ) in nm.

cluster num by count	hdbscan id	cluster size	% K30	% K41	% K47	% K51	% K56	% K63	% of combined ensemble	μ ISA score	σ ISA score	μ internal rmsd in nm	σ internal rmsd in nm
1	18	8557			0.0	98.4	1.6		1.29	92.94	69.19	0.47	0.32
2	3	5230		0.1				99.9	0.79	185.75	102.45	0.29	0.07
4	46	4461	0.7		0.4	11.0	2.3	85.7	0.67	98.80	33.80	0.45	0.41
5	16	3897		10.3	19.2	1.8		68.8	0.59	159.18	85.53	0.41	0.19
6	28	3362			92.2	7.8			0.51	63.30	56.19	0.39	0.10
7	1	3327			93.9		6.1		0.50	120.34	47.36	0.27	0.15
8	43	3242	9.9	87.0	0.4		2.7		0.49	93.50	32.32	0.44	0.18
9	39	2463						100.0	0.37	182.25	101.69	0.29	0.07
10	13	2266			83.1	14.4	2.4	0.0	0.34	130.23	56.89	0.37	0.29
11	172	2174		0.1		75.4	24.5		0.33	66.00	21.73	0.48	0.27
12	23	1852	98.5	1.5					0.28	23.33	8.16	0.26	0.06
13	14	1821			95.4	4.6			0.27	72.56	55.45	0.33	0.12
14	15	1670			85.1	6.5		8.4	0.25	66.92	32.01	0.41	0.20
15	90	1658	5.9	2.0	1.7	0.1	4.0	86.2	0.25	76.23	43.33	0.37	0.36
16	7	1476			94.0	6.0		0.1	0.22	70.57	32.02	0.35	0.14
17	5	1408			100.0				0.21	176.00	87.00	0.29	0.06
18	2	1305			1.2	98.8			0.20	88.06	94.67	0.41	0.10
19	33	1301			0.1	99.9			0.20	49.69	50.08	0.35	0.14
20	0	1221	100.0						0.18	21.50	7.14	0.27	0.13
21	53	1131	0.1		99.9				0.17	21.99	5.77	0.39	0.09
22	175	1067	27.3		0.4	4.3		68.0	0.16	67.49	36.16	0.56	0.29
23	21	1003			100.0				0.15	26.54	7.75	0.26	0.08
24	11	1003						100.0	0.15	31.47	13.57	0.35	0.13
25	4	996			100.0				0.15	46.76	14.14	0.36	0.08
26	40	940			1.0	98.7	0.3		0.14	91.85	68.76	0.33	0.14
27	22	835			87.1	9.3	3.5	0.1	0.13	130.93	59.50	0.32	0.21
28	17	823	0.2		99.6			0.1	0.12	123.89	66.86	0.22	0.06
29	8	805	1.0		99.0				0.12	22.25	5.85	0.40	0.12
30	10	790						100.0	0.12	134.32	82.36	0.26	0.04
31	6	767			96.5	3.5			0.12	68.50	26.54	0.33	0.12
32	12	720			100.0				0.11	127.23	55.93	0.22	0.04
33	455	689			99.1		0.9		0.10	48.44	10.95	0.34	0.09
34	194	688	84.7		11.5	0.4	3.3		0.10	94.12	100.33	0.31	0.11
35	65	684	96.9	2.8	0.1			0.1	0.10	119.83	27.21	0.45	0.17
36	44	679	1.5	0.1	0.3	0.1	3.5	94.4	0.10	73.12	37.40	0.26	0.27
37	31	678						100.0	0.10	146.05	82.32	0.24	0.05
38	351	654			1.2	73.7	6.4	18.7	0.10	41.83	33.75	0.52	0.15
39	45	644	6.5		0.5	12.6		80.4	0.10	49.01	24.83	0.39	0.27
40	121	606		2.3	1.0		96.7		0.09	74.23	17.69	0.45	0.25
41	9	588			100.0				0.09	33.26	10.60	0.31	0.11
42	36	588	0.7				0.7	98.6	0.09	94.36	49.96	0.27	0.14
43	441	580		100.0					0.09	20.75	4.29	0.25	0.05
44	109	573	49.0		33.2	17.8			0.09	93.57	67.11	0.48	0.22
45	68	544		2.0	13.6		84.4		0.08	74.47	91.27	0.46	0.16
46	35	541						100.0	0.08	76.08	38.35	0.21	0.06
47	274	532			73.1	7.0	10.5	9.4	0.08	79.31	77.38	0.46	0.37
48	24	505	2.0	14.3	83.8				0.08	18.60	3.29	0.33	0.15
49	116	486			100.0				0.07	91.48	91.94	0.41	0.17
50	56	475	0.2	0.4		4.8		94.5	0.07	172.05	95.63	0.35	0.17
51	20	447	100.0						0.07	41.01	32.95	0.26	0.06
