# Supplementary material for: Combining molecular dynamics simulations and scoring method to computationally model ubiquitylated linker histones in chromatosomes
Source: PLoS Comput Biol. 2023 Aug 1;19(8):e1010531. doi: 10.1371/journal.pcbi.1010531 (PMC10442151; doi:10.1371/journal.pcbi.1010531)
Supplement: S2 Table — All references to a score mean the ISA score. Clusters were selected by choosing clusters with the lowest mean ISA score and at least 90% composition of the respective ubiquitylation sit. (PDF) [file pcbi.1010531.s005.pdf]

Table S2: Overview over the composition and scores of the ubiquitylation sites. All references to a score mean the ISA score. Clusters were selected by choosing clusters with the lowest mean ISA score and at least 90% composition of the respective ubiquitylation site.

ubq site	total frames	frames in clusters	% clustered	% not clustered	µ ISA scores 4QLC	µ ISA scores 5NL0	µ ISA scores 5WCU	µ ISA scores all	µ ISA scores off dyad	σ ISA scores 4QLC	σ ISA scores 5NL0	σ ISA scores 5WCU	σ ISA scores all	σ ISA scores off dyad
K30	79398	13383	16.86	83.14	74.46	85.44	75.17	74.46	28.39	53.67	59.49	57.94	53.67	36.67
K41	79398	19084	24.04	75.96	74.68	91.39	77.36	74.68	78.28	50.28	61.92	56.18	50.28	68.17
K47	144201	48224	33.44	66.56	75.99	86.56	79.26	75.99	98.12	59.66	71.22	65.97	59.66	76.67
K51	139401	35216	25.26	74.74	74.03	75.74	76.30	74.03	112.96	61.88	59.37	66.01	61.88	83.03
K56	79398	12656	15.94	84.06	41.15	48.48	40.91	41.15	140.43	24.44	30.12	25.85	24.44	75.97
K63	140937	38645	27.42	72.58	105.37	111.09	106.95	105.37	48.40	68.62	77.38	73.09	68.62	50.28
