# Supplementary material for: Combining molecular dynamics simulations and scoring method to computationally model ubiquitylated linker histones in chromatosomes
Source: PLoS Comput Biol. 2023 Aug 1;19(8):e1010531. doi: 10.1371/journal.pcbi.1010531 (PMC10442151; doi:10.1371/journal.pcbi.1010531)
Supplement: S3 Table — The reference chromatosomes pdb 5NL0 and the off-dyad structure were manually adjusted to allow their linker histone to fit into the tetranucleosome array of pdb 1ZBB. In the following table these adjustments are listed. “Core” refers to one of the cores of 1ZBB, where core1 has the lower segid of the first frame of the biological assembly and core4 has the higher segid of the second frame of the biological assembly. (PDF) [file pcbi.1010531.s006.pdf]

Table S3: Manual adjustments to create hypothetical linker histone positions in the tetranucleosome array 1ZBB. The reference chromatosomes pdb 5NL0 and the off-dyad structure were manually adjusted to allow their linker histone to fit into the tetranucleosome array of pdb 1ZBB. In the following table these adjustments are listed. “Core” refers to one of the cores of 1ZBB, where core1 has the lower segid of the first frame of the biological assembly and core4 has the higher segid of the second frame of the biological assembly.

Alignment	origin	Adjustments made
5NL0 to core 1 of 1ZBB	1ZBB	rotated 5NL0 5 deg around third principal axis
5NL0 to core 2 of 1ZBB	1ZBB	rotated 5NL0 31 deg around third principal axis
5NL0 to core 3 of 1ZBB	1ZBB_frame_2	rotated 5NL0 -5 deg around third principal axis
5NL0 to core 4 of 1ZBB	1ZBB_frame_2	rotated 5NL0 -30 deg around first principal axis
off-dyad to core 1 of 1ZBB	1ZBB	No additional adjustemts made
off-dyad to core 2 of 1ZBB	1ZBB	-15 deg around third principal axis
off-dyad to core 3 of 1ZBB	1ZBB_frame_2	No additional adjustment made
off-dyad to core 4 of 1ZBB	1ZBB_frame_2	8 deg around third principal axis
