# Supplementary figures and images for: Combining molecular dynamics simulations and scoring method to computationally model ubiquitylated linker histones in chromatosomes
Source: PLoS Comput Biol. 2023 Aug 1;19(8):e1010531. doi: 10.1371/journal.pcbi.1010531 (PMC10442151; doi:10.1371/journal.pcbi.1010531)

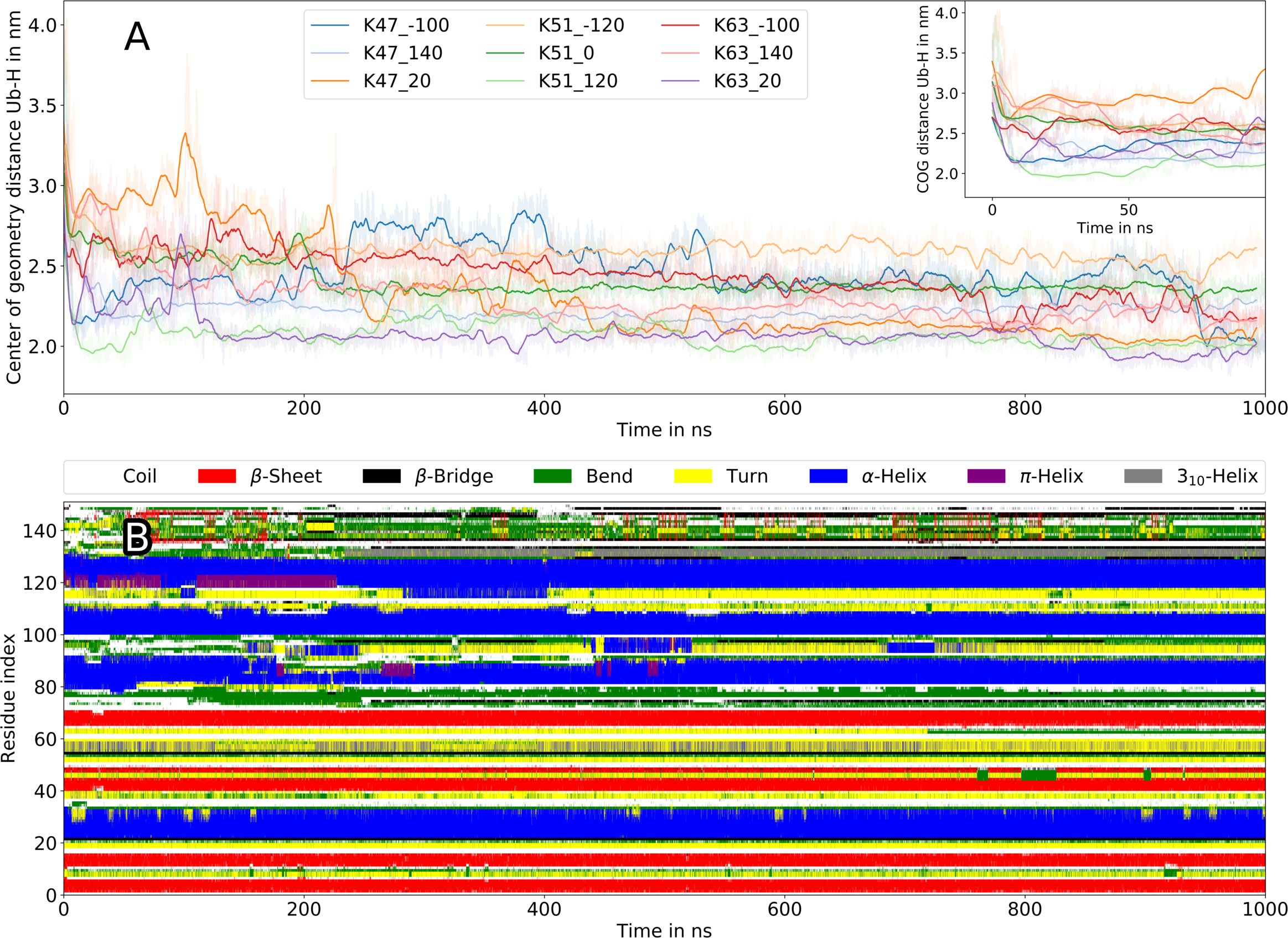

Supplement: S1 Fig — Time evolution of the center of geometry distances between the Ub-subunit and the H1-subunit of nine 1 μs simulations (A). Simulation names are composed of ubiquitylated lysine and starting χ3 angle (KXXX_χ3). The inset figure shows the same data in a 0–100 ns interval. Raw distances are transparent. Running averages over 7.5 ns are opaque. After relaxation (until ≈ 25 ns) the center of geometry stays similar during the evolution of the whole simulation. K47Ub with χ3 = 20° exhibits larger variations at the beginning, but approaches an equilibrium after that. The secondary structure motifs of this simulation also exhibit convergence of the structure (B). (TIF) [file pcbi.1010531.s007.tif]

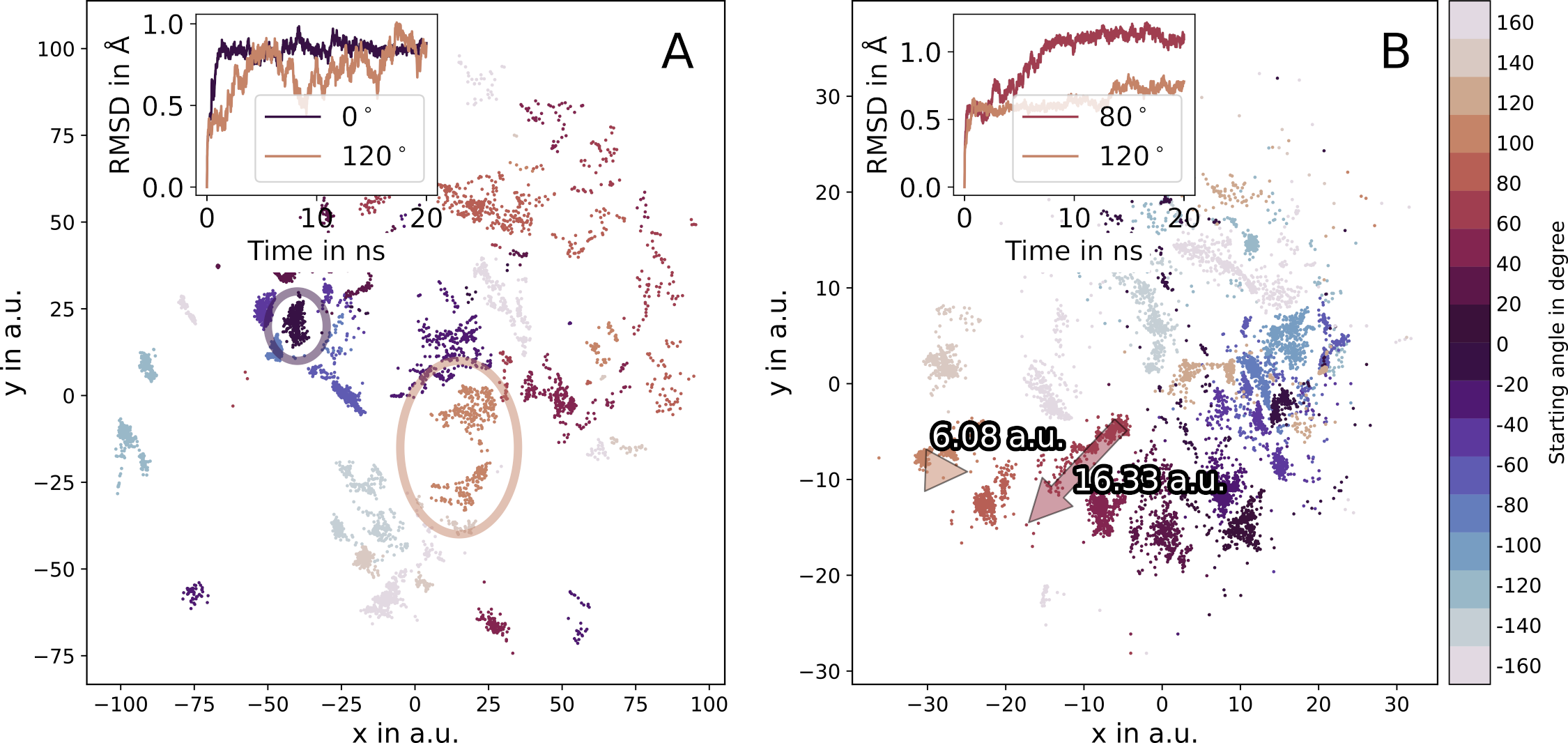

Supplement: S2 Fig — Exemplary sketch-map projections of initial simulations of K47Ub using SASA-CVs (A) and K30Ub using RMD-CVs (B). The color code indicates the starting χ3 angle of the respective ubiquitylated lysine residue. Similar angles have similar color saturation. Inset figures show RMSD evolution of selected trajectories. Larger RMSD deviations result in disjoint, scattered points. Smaller RMSD deviations yield cohesive patches. The simulation of K47Ub with a starting angle of χ3 = 0° exhibits little change in its RMSD after relaxation, which results in a densely populated patch (circle in (A)). Arrows in and annotated distances (B) connect the first and last point of the simulations with starting angle χ3 = 80° and χ3 = 120° and indicate the time evolution of these trajectories. The RMSD of the structures from K30Ub with an angle of χ3 = 120° exhibits a gradual increase, which is also traced in the sketch-map projection. (TIF) [file pcbi.1010531.s008.tif]

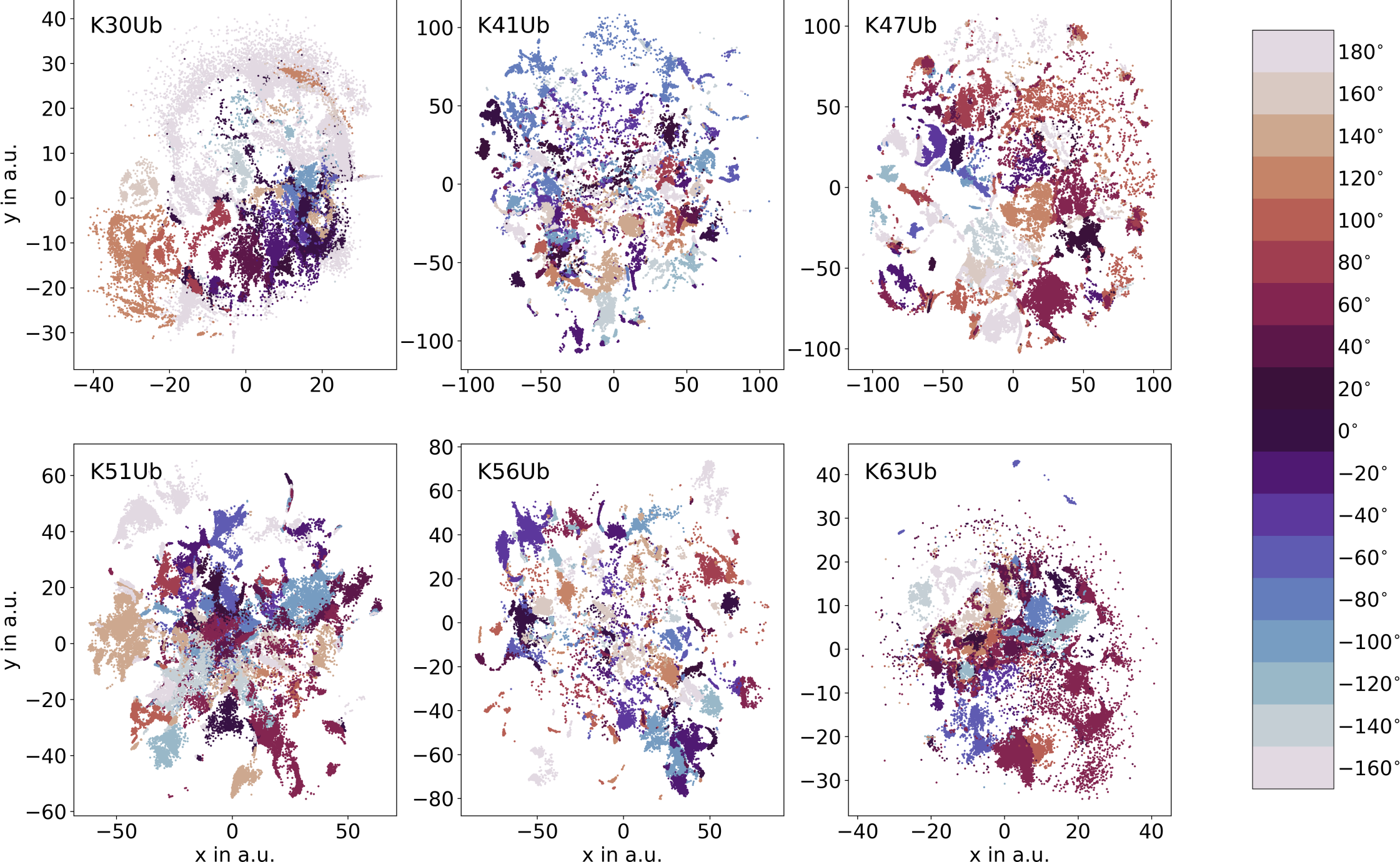

Supplement: S3 Fig — K47Ub and K51Ub were projected using the SASA-CVs (Eq (1)) as input CVs for sketch-map. The other 4 variants were projected using the RMD-CVs (Eq (2)). Points are colored according to starting χ3 angle of the ubiquitylated lysine residue. A cyclic colormap was chosen to represent the periodic nature of dihedral angles. (TIF) [file pcbi.1010531.s009.tif]

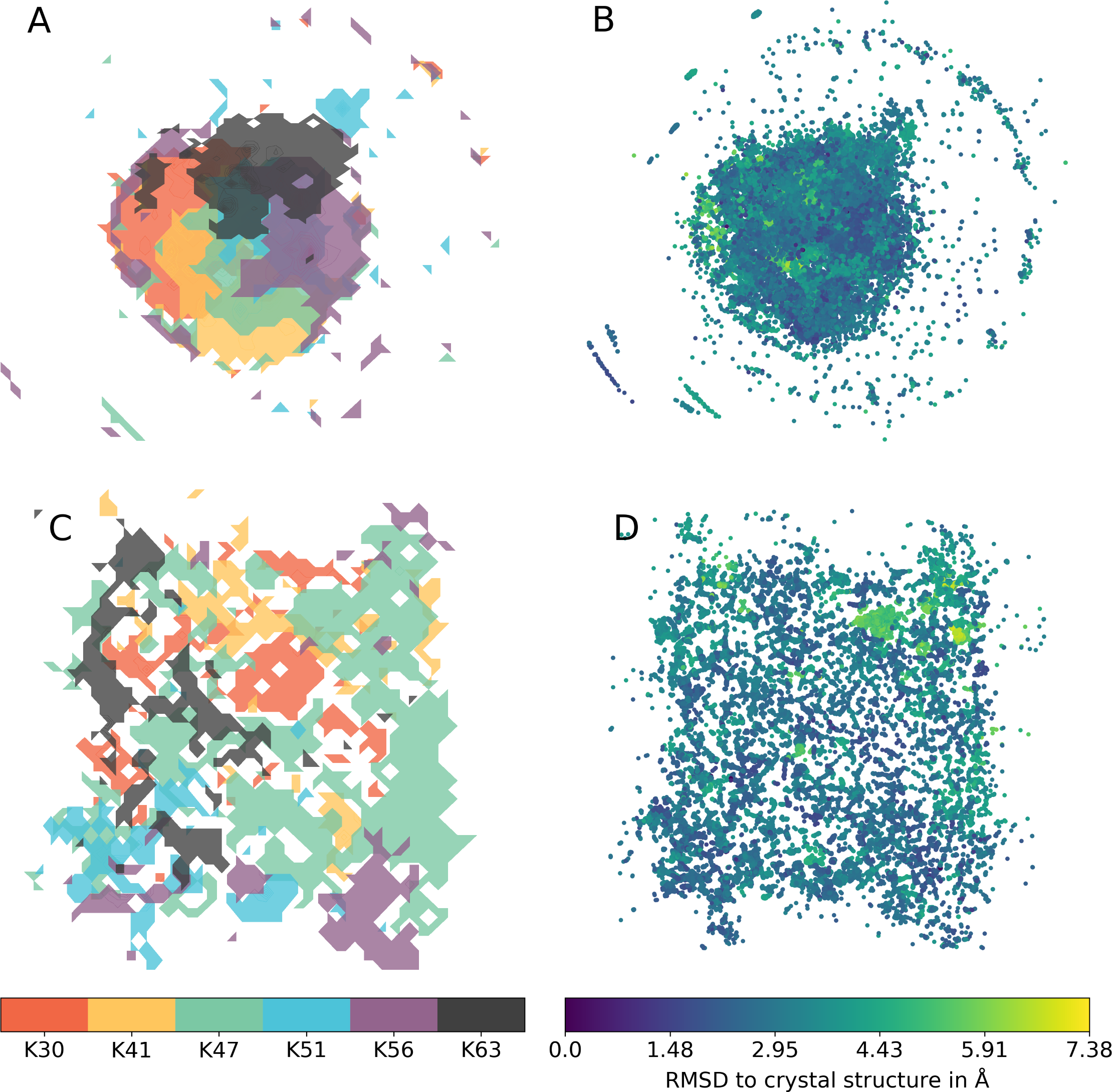

Supplement: S4 Fig — For the top images (A-B) the RMD-CVs have been used as high-dimensional input for sketch-map. The lower images (C-D) were created by projecting the SASA-CVs. (A) and (C) are colored according to the ubiquitylation site of the variant. (B) and (D) are colored according to the mean RMSD distance to the subunit’s respective crystal structure (PDB ID 1GHC and 1UBQ). The RMD-CVs are not as suited as the SASA-CVs to project the conformational space of the six HUb proteins into the same low-dimensional map. More disorder for the top images can be observed than the for the bottom images, which contained the SASA-CVs in their creation. Thus the SASA-CVs were chosen to use as the high-dimensional collective variables for projecting the conformational space of all six variants into the same low-dimensional map. (TIF) [file pcbi.1010531.s010.tif]

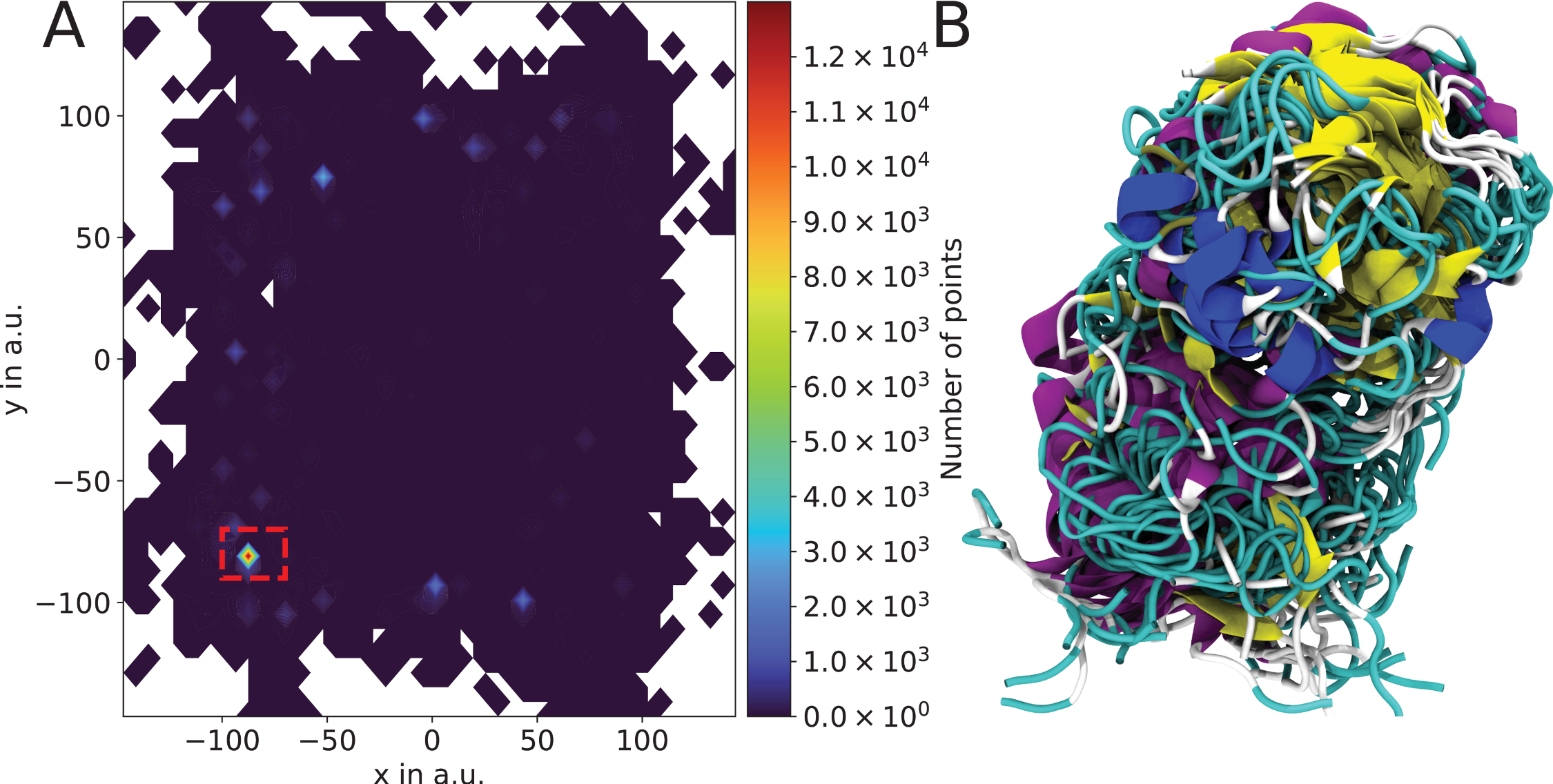

Supplement: S5 Fig — In (A) the low-dimensional sketch-map projections (x and y coordinates) of all six variants using the SASA-CVs (Eq (1)) were plotted as a density map (colormap on the right). The high-density region is highlighted with a rectangle. In (B) 100 protein conformations originating from this region are shown. sketch-map was not able to separate these different conformations or even push them to the fringes of the projection map. For further analyses this region was excluded. (TIF) [file pcbi.1010531.s011.tif]

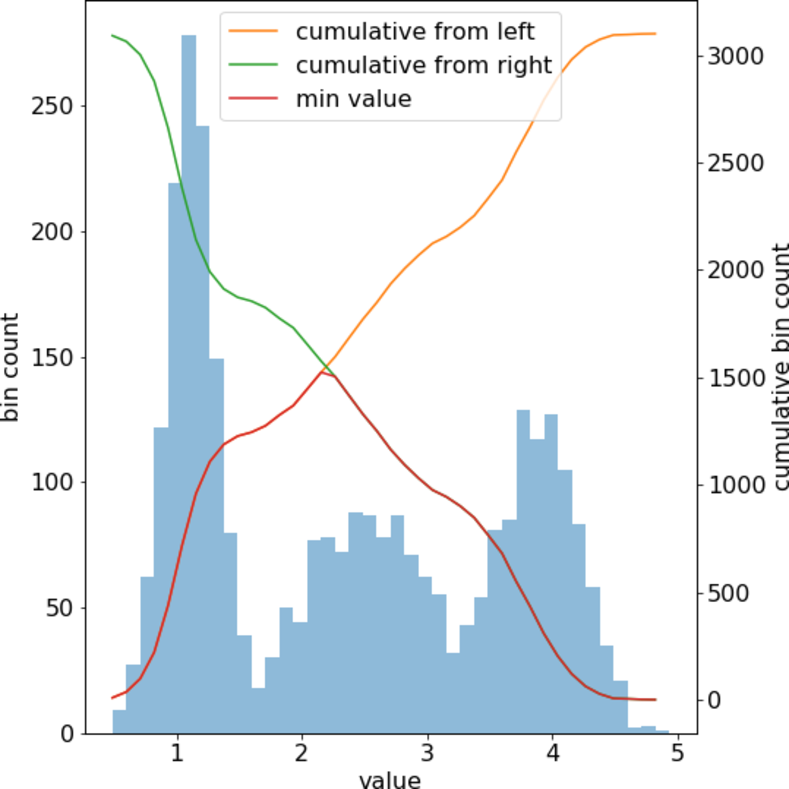

Supplement: S6 Fig — The 1D bounding box ranges from 0 to 5. Two cumulative histograms are created from a base histogram (blue). One ascending (orange), the other one descending (green). Both are visualized as smoothed curves. The final histogram which is then used for scoring is obtained by choosing the minimal value of either of the two cumulative histograms (red). (TIF) [file pcbi.1010531.s012.tif]

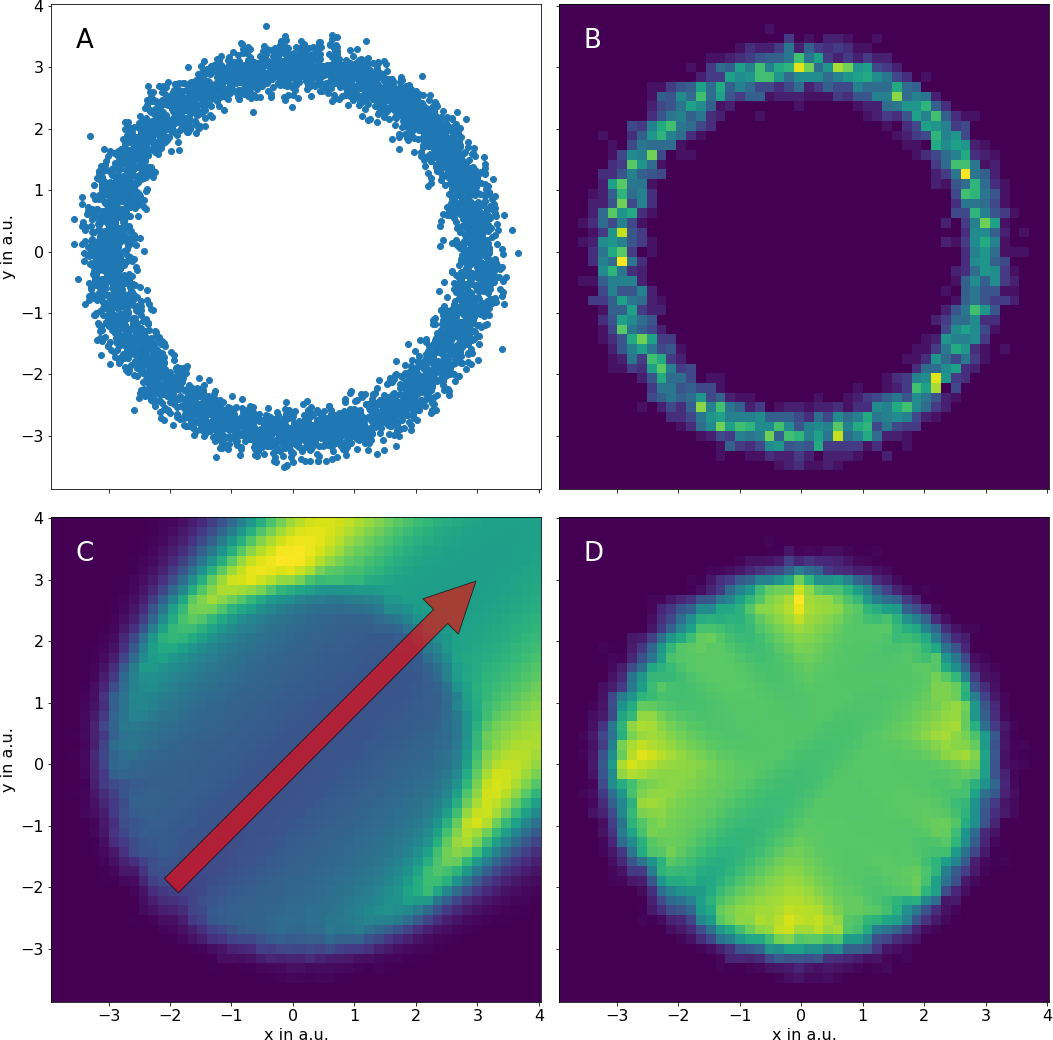

Supplement: S7 Fig — In (A) 500 points have been placed in a ring with radius r = 3 ± N(μ = 0, σ = 0.2), where N is a standard normal distribution, around the origin. A normal histogram counting the number of points in a predefined set of bins is shown in (B), where yellow denotes highly occupied bins, purple empty bins. In (C) one of the four histograms obtained by walking from the lower left corner to the upper right is displayed. This results in some smoothing and “smearing” to the top right. In (D) the histogram for scoring is constructed by choosing the lowest value from the 4 cumulative histograms per bin. This histogram is used in ISA to determine the score of a given HUb-chromatosome pose. (TIF) [file pcbi.1010531.s013.tif]

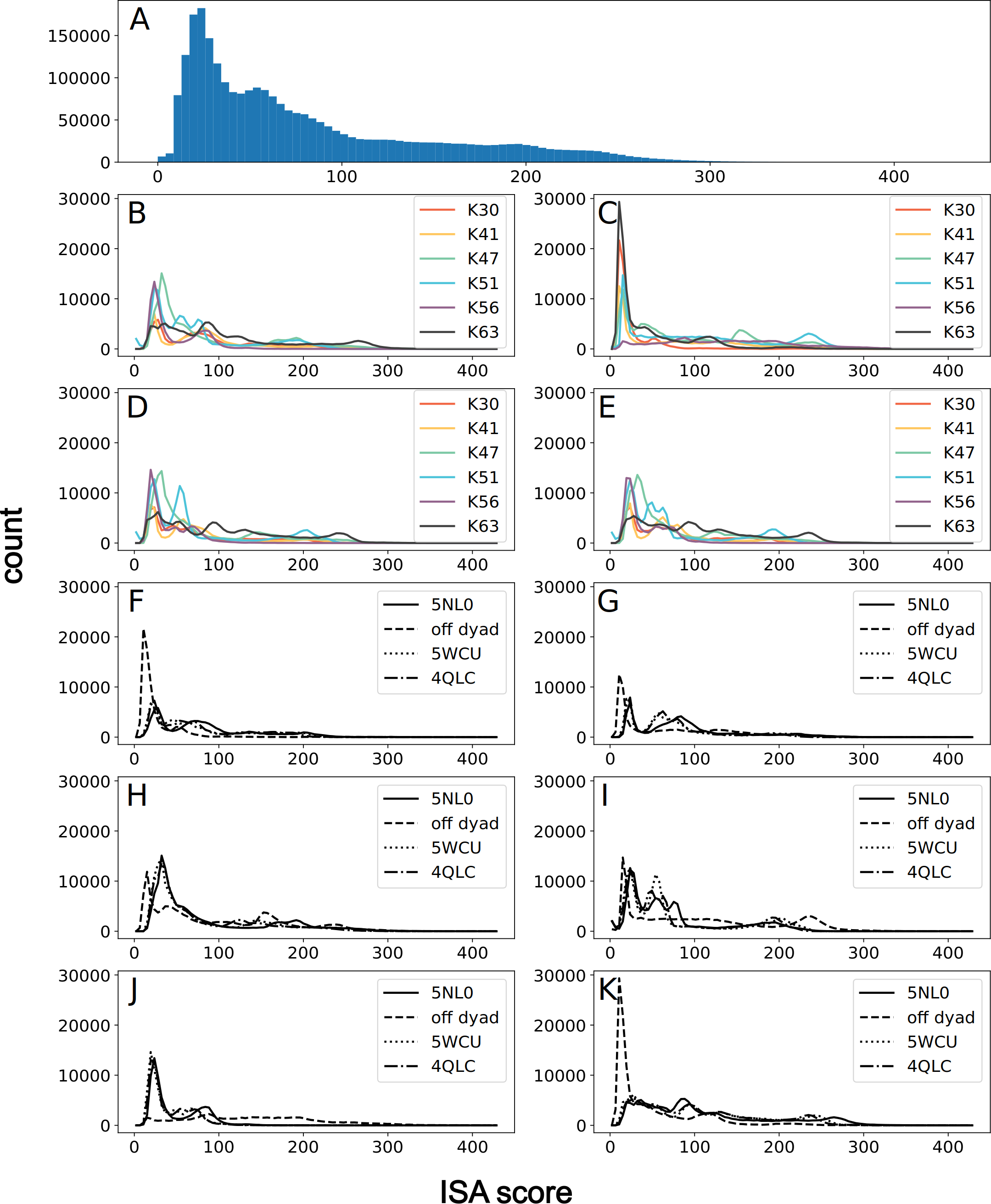

Supplement: S8 Fig — The simulations of the six linkage types (K30 to K63) were placed into the four parent chromatosomes (5NL0, 5WCU, 4QLC and the off-dyad structure). In (A) the scores of all poses are visualized. The count reaches as high as 150 000 because all ubiquitylation variants and chromatosomes are combined. The four subfigures (B-E) give the per ubiquitylation variant score for the four chromatosomes 4QLC, 5NL0, 5WCU, and off-dyad respectively. It can be seen, that K56Ub (purple) tends to display higher scores after being placed into the off-dyad chromatosome (E) than for the 4QLC chromatosome (B). The remaining six subfigures (F-K) give the per chromatosome score for the six ubiquitylation variants K30Ub, K41Ub, K47Ub, K51Ub, K56Ub, and K63Ub, respectively. Here, it can be seen that the scores for the chromatosomes 5WCU and 4QLC (green and dark purple) are very similar. They almost coincide for all ubiquitylation variants. Furthermore, K56Ub (is very unfavorably positioned for the off-dyad chromatosome (yellow). (TIF) [file pcbi.1010531.s014.tif]

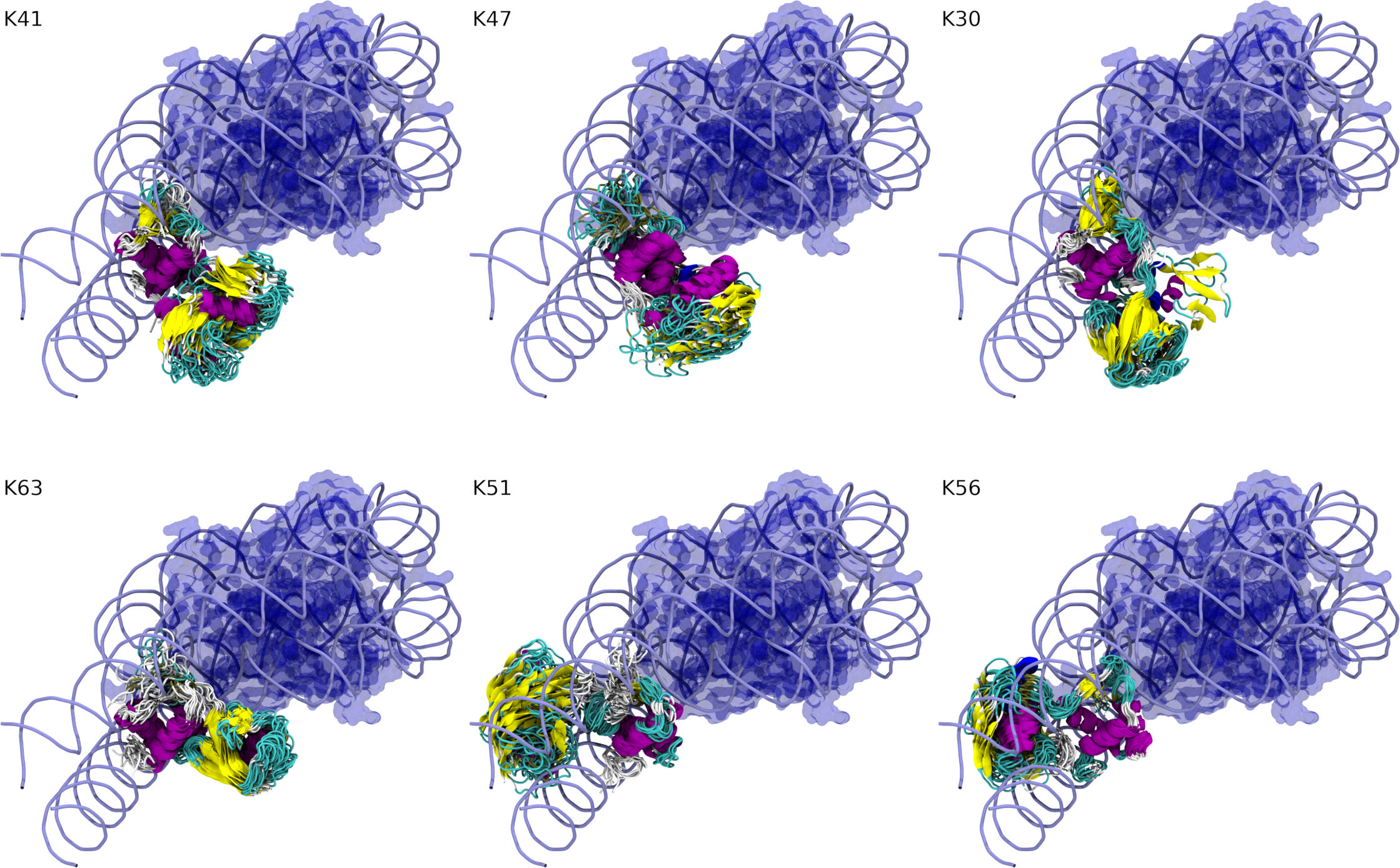

Supplement: S9 Fig — From all clusters found by iterative HDBSCAN clustering the cluster with the lowest ISA score containing at least 90% of a specific HUb variant is chosen. 20 equally spaced frames of these clusters are then placed into the parent 5NL0 chromatosome structure and visualized here. All of them have the Ub subunit pointing downward. For scores of other clusters refer to S1 Table. (TIF) [file pcbi.1010531.s015.tif]
